# Antimicrobial potential of bacteria isolated from sediment and *Avicennia germinans* collected from the mangroves of Vichayal (Northern Peru, Piura) and their application in the preservation of *Scomber japonicus peruanus*

**DOI:** 10.3389/fmicb.2026.1879875

**Published:** 2026-06-24

**Authors:** Edwin Jorge Vega-Portalatino, Miriam Marleni Rosales-Cuentas, Carlos Javier Rojas-Espinoza, Heber Peleg Cornelio-Santiago, Jaime Valdiviezo-Marcelo, Luz Arelis Moreno-Quispe

**Affiliations:** 1 Laboratorio de Investigación en Biotecnología Microbiana, Universidad Nacional de Frontera, Sullana, Peru; 2 Laboratorio de Análisis de Agua y Suelo, Universidad Nacional de Frontera, Sullana, Piura, Peru; 3 Facultad de Ciencias de los Alimentos, Universidad Le Cordon Bleu, Lima, Peru; 4 Grupo de Investigación Tecnologías Emergentes, Investigaciones de Mercado y Comportamiento del Consumidor (TEIMEC), Universidad Nacional de Frontera, Sullana, Peru; 5 Escuela de Administración de Empresas, Universidad Nacional de Barranca, Lima, Peru

**Keywords:** bacteria, fish, inhibition, pathogens, yeasts

## Abstract

Mangrove ecosystems in Peru represent largely unexplored reservoirs of bacterial diversity with considerable biotechnological potential. This study evaluated the antimicrobial activity of bacteria isolated from sediments and endophytic tissues of *Avicennia germinans* from the Vichayal mangroves (Piura, northern Peru) using the volatile organic compounds test (VOCt), the Over Plate Test (OpT), minimum inhibitory concentration (MIC), and minimum bactericidal concentration (MBC) against Gram-positive pathogenic bacteria (*Enterococcus faecalis*, *Staphylococcus epidermidis*, *S. aureus*, and *Listeria monocytogenes*), Gram-negative pathogenic bacteria (*Escherichia coli* and *Salmonella enterica* sv. *typhimurium*), and two *Candida* species (*Candida albicans* and *C. tropicalis*), as well as their application in the preservation of *Scomber japonicus* fillets, prior to biosafety testing and phylogenetic analysis. A total of 34 isolates were obtained — 21 from sediments and 13 from *A. germinans*. Based on the VOCt and OpT assays, seven strains were identified by 16S rDNA sequencing as *Bacillus albus* ZB3, *Priestia flexa* (ZA5 and ZA9), *Enterococcus faecalis* ZB5, *Pseudomonas monteilii* (ZA1 and ZAAgR1), and *Acinetobacter junii* ZB7, yielding cell-free extracts containing bioactive substances (42.95–47.44 mg/mL). MIC results showed that 85.7% of the strains inhibited *S. aureus* ATCC25923 and *E. coli* O157:H7, while MBC activity was confirmed for ZA1, ZB3, and ZAAgR1 at extract concentrations ≥10%. The biosafety profile revealed gamma-hemolysis and the absence of DNase activity across all strains, consistent with a non-virulent phenotype; however, widespread antibiotic resistance was detected. Application of cell-free extracts to mackerel fillets demonstrated that *B. albus* ZB3 reduced aerobic mesophile counts (6.19 vs. 6.46 log₁₀ CFU/g) at ambient temperature (27 ± 2 °C, 2 days), while *P. monteilii* ZAAgR1 was more effective under refrigeration (6.19 vs. 6.36 log₁₀ CFU/g, 7 ± 2 °C, 7 days). These findings identify bacteria associated with the Vichayal mangroves as promising candidates for fishery product preservation, pending comprehensive genomic and safety characterization.

## 1 Introduction

*Scomber japonicus peruanus* is one of the most commercially important fishery resources of the South Pacific and a key component of food security in Peru (Torrejón-Magallanes et al., 2017; Arevalo and Castro, 2022). Nevertheless, its high proportion of nitrogenous compounds, unsaturated lipids, and enzymatic activity accelerates post-mortem spoilage processes, including lipid oxidation, proteolysis, and microbial proliferation, which compromise its sensory quality and nutritional value (Jeong-seok et al., 2023; Soodsawaeng et al., 2022; Torrejón-Magallanes et al., 2017; Miranda et al., 2022; El-tahlawy et al., 2026).

The dynamics of microbial spoilage in fish are modulated by intrinsic and extrinsic factors, such as initial microbiota, environmental conditions, handling practices, and storage temperature (Altayr et al., 2025; Sheng and Wang, 2021). In this context, microorganisms such as *Pseudomonas* spp., *Enterococcus* spp., and *Salmonella* spp., along with specific psychrotrophic spoilage bacteria, play a determining role in reducing shelf life and in the risks associated with food safety (El-tahlawy et al., 2026).

In response to these limitations, the development of biopreservation strategies based on natural compounds has attracted increasing interest as an alternative to synthetic preservatives, whose safety and consumer acceptance are increasingly questioned (Yazgan et al., 2024; Jeong-seok et al., 2023; El-tahlawy et al., 2026). In particular, mangrove ecosystems represent largely unexplored reservoirs of microbial diversity (Sandoval and Marcial, 2022) with high biotechnological potential. Microbial associated with sediments or plant tissues of *Avicennia germinans* have demonstrated the capacity to synthesize secondary metabolites with antimicrobial properties (Liu et al., 2026; Lemos-lucumi et al., 2025); however, their application in food matrices, especially in fishery products, remains limited and poorly characterized.

These metabolites—including polyphenols, organic acids, and/or bacteriocins—can exert their action through multiple mechanisms, such as disruption of cell membrane integrity, interference with the electron transport chain, and inhibition of essential macromolecule synthesis (Lemos-lucumi et al., 2025; Wang and Fang, 2026), which confers upon them a broad spectrum of activity against pathogenic and spoilage microorganisms (Vega-portalatino et al., 2024; Vega-portalatino et al., 2023).

Despite this potential, a significant gap remains in the evaluation of mangrove-derived bacteria as biopreservative agents applied directly in real food systems, particularly in species of high commercial relevance such as *S. japonicus peruanus*. In this context, we propose the hypothesis that bacteria isolated from sediments and endophytes of *A. germinans* produce metabolites with antimicrobial activity capable of inhibiting pathogenic and spoilage microorganisms, with potential application in the preservation of the microbiological and physicochemical quality of mackerel fillets during storage.

More specifically, it is proposed that: (i) the isolated bacteria exhibit significant antimicrobial activity against pathogenic microorganisms, as evidenced by *in vitro* assays; (ii) this activity is associated with the production of bioactive substances with mechanisms of action targeting microbial cell integrity and function; (iii) the application of metabolites from selected strains—following biosafety testing (hemolytic activity, DNase production, and antibiotic susceptibility)—to *S. japonicus peruanus* fillets reduces microbial load and delays spoilage; and (iv) these treatments have the potential to preserve physicochemical parameters related to freshness during storage.

Accordingly, the present study aimed to evaluate the antimicrobial potential of bacteria isolated from sediments and endophytes of *A. germinans* from the Vichayal mangroves (Piura, Peru), as well as to assess biosafety parameters (hemolytic activity, DNase production, and antibiotic susceptibility) and to explore their potential application in the preservation of the microbiological and physicochemical quality of *S. japonicus peruanus* fillets during storage.

## 2 Materials and methods

### 2.1 Sample collection

**Figure 1:**
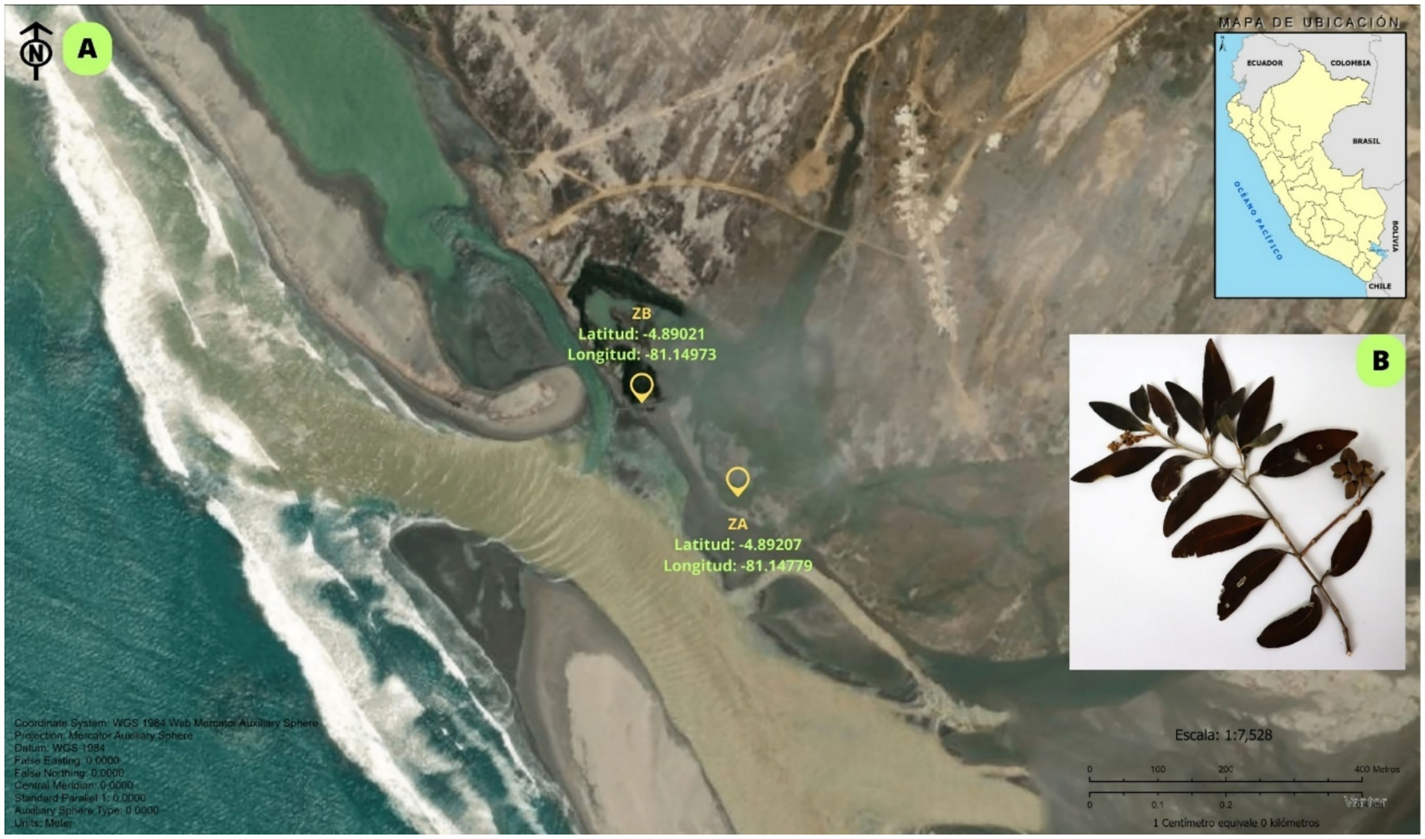
Collection site of sediment and *Avicennia germinans*, Vichayal Mangroves, Paita, Piura, Peru. **(A)** Collection site, ZA (−4.89207, −81.14779) and ZB (−4.89021, −81.14973) (Software: National Geographic Institute of Peru – Center for Geospatial Data Infrastructure). **(B)** Specimen of *Avicennia germinans*.

One hundred grams of sediment from the Vichayal mangroves were collected using a sterile shovel at a depth of 10 cm from three sampling points spaced 5 meters apart, under aseptic conditions, within the district of La Bocana, Paita, Piura, Peru, located in Zone A (−4.89207, −81.14779) and Zone B (−4.89021, −81.14973), as shown in Figure 1. With respect to *Avicennia germinans*, five samples (leaves, stems, and roots) were collected from three plants showing no signs of disease or external damage, from the previously referenced zones. All samples were transferred to sterile containers and transported to the Microbial Biotechnology Research Laboratory of the Universidad Nacional de Frontera under aseptic conditions, and stored at 4 °C for 24 h. The taxonomic identification of the plant specimen was performed through herbarium preparation and identification at the Herbarium Truxillense (HUT) of the Universidad Nacional de Trujillo (Trujillo, Peru), under registration code 66114.

Additionally, physicochemical water parameters (Table 1) were measured, including temperature (T), hydrogen potential (pH), dissolved oxygen (DO), and electrical conductivity (EC), using a multiparameter water meter (HANNA Instruments, Romania). Hardness (Ha), chlorides (Cl), alkalinity (Alk), and turbidity (Tu) were additionally determined at the Water and Soil Analysis Laboratory of the Universidad Nacional de Frontera.

**Table 1:** Physicochemical water parameters at the collection points of the Vichayal Mangroves.

Collection point	T (°C)	pH	OD (ppm)	Ce (μS/cm)	Du (CaCO_3_/L)	Cl (mg Cl^−^/L)	Alc (CaCO_3_/L)	Tu (NTU)
ZA	22.2	7.77	25.16	32,900	106.1	19.4	136.6	5.07
ZB	23.6	7.31	18.8	36,600	114.3	19.4	142.8	8.55

T: temperature; pH: hydrogen potential; DO: dissolved oxygen; EC: electrical conduc-tivity; Ha: hardness; Cl: chlorides; Alk: alkalinity; Tu: turbidity.

### 2.2 Conditioning, isolation, and selection of bacteria

Three hundred grams of sediment per collection zone were pooled and three 15 g subsamples were extracted; each subsample was transferred to flasks containing 150 mL of 0.8% NaCl (w/v). Samples were homogenized for 30 min at 700 rpm using an orbital shaker. Isolation was performed using the serial dilution technique proposed by Li et al. (2023) and Agurto-Fabiola et al. (2025), with some modifications; 100 μL of the 10^−4^ dilution were inoculated by surface spreading onto plates containing tryptic soy agar (TSA) supplemented with nystatin (100 μg/mL). Plates were incubated at 25 °C for 24 h. Regarding *Avicennia germinans*, thirty leaves, stems, and roots were washed with running water to remove external residues. Subsequently, thirty fragments (1.5 cm) were cut and disinfected in a beaker with 0.1% mercuric dichloride, followed by 5 successive washes with sterile distilled water for 5 min each, per fragment type. The disinfected fragments were placed in vials containing 2 mL of tryptic soy broth (TSB) for 5 min as a negative control. They were then removed from the vials, transversally cut into two pieces on Petri dishes with sterile absorbent paper, and placed into vials containing 2 mL of TSA supplemented with nystatin (100 μg/mL). The fragments and their disinfection controls were incubated at 25 °C for 7 days. Bacterial growth around the fragment, combined with the absence of bacterial growth in the negative control, was considered indicative of endophytic origin; this methodology was described by Vega-portalatino et al. (2024), with some modifications.

The isolated bacteria were purified by successive streaking onto TSA plates until axenic colonies were obtained, and were maintained in test tubes with slanted TSA. All strains were cryopreserved in TSB-glycerol medium at 30%.

### 2.3 Antibacterial and anti-Candida activity

#### 2.3.1 Pathogenic bacteria and Candida species

The isolated bacteria were evaluated against 4 Gram-positive bacteria (*E. faecalis* ATCC29212, *S. epidermidis* ATCC12228, *S. aureus* ATCC25923, *S. aureus* ATCC29213, *S. aureus* ATCC700699, and *L. monocytogenes* ATCC7644), 3 Gram-negative bacteria (*E. coli* O157: H7, *E. coli* ATCC10536, and *S. enterica* sv. *typhimurium* ATCC14028), and 2 Candida species (*Candida albicans* ATCC90028 and *C. tropicalis* ATCC750).

#### 2.3.2 Inoculum preparation

Isolated bacteria and pathogenic bacteria were cultured in TSB (1 mL), while yeasts were cultured in potato dextrose broth (PDB) (1 mL) and incubated at 25 °C; cultures were then centrifuged at 2,000 × g for 5 min and the pellet was resuspended in 0.8% NaCl to an optical density of 0.08 at 620 nm, according to Vega-portalatino et al. (2024).

#### 2.3.3 Volatile organic compounds test (VOCt)

The assay was performed as described by Vega-portalatino et al. (2024). One hundred microliters of the isolated bacteria (OD₆₂₀: 0.08) were inoculated by surface spreading onto TSA plates. In parallel, 3 filter paper discs (6 mm) impregnated with 2 μL of pathogenic bacteria were placed on Mueller-Hinton agar (MHA) plates, and 2 μL of pathogenic yeasts were inoculated onto PDA plates. Both plates were joined face-to-face and sealed with Parafilm, with the isolated bacteria on the bottom and the pathogens on top. TSA plates with pathogen-impregnated discs served as growth controls; all plates were incubated at 25 °C for 2 to 4 days. Growth inhibition of pathogens was expressed as percentage inhibition (AH%) = [(A − B)/A] × 100, where A is the diameter of the pathogen without the isolated bacterium and B is the diameter of the pathogen in the presence of the isolated bacterium.

#### 2.3.4 Ensayo over plate test (OpT)

One hundred microliters of isolated bacteria (OD₆₂₀: 0.08) were incorporated into 20 mL of TSA at fusion temperature (40 °C), poured onto Petri dishes, and incubated at 25 °C for 14 days to induce the production of antimicrobial metabolites. Cultures were then cut into 5 mm discs. For the OpT, 100 μL of pathogenic bacteria and yeasts (OD₆₂₀: 0.08) were inoculated by surface spreading onto TSA and PDA plates, respectively, and 3 discs of the isolated bacteria were immediately placed on the surface, followed by incubation at 25 °C for 24 h, according to Vega-portalatino et al. (2024). TSA discs (5 mm) served as negative controls. Clear inhibition zones indicated antibacterial activity and were expressed in millimeters (mm).

#### 2.3.5 Minimum inhibitory concentration (MIC) and minimum bactericidal concentration (MBC)

Seven strains with the highest antimicrobial activity from the VOCt and OpT assays were selected for the minimum inhibitory concentration (MIC) assay, following the methodology described by Tamariz-angeles et al. (2023) and Vega-portalatino et al. (2024). The selected bacteria (OD₆₂₀: 0.08) were inoculated into TSB (50 mL) and incubated at ambient temperature (27 ± 2 °C) on an orbital shaker for 14 days. The cultures were then centrifuged at 2,000 × g for 15 min to obtain the supernatant, which was subsequently filtered through a Millipore membrane (0.22 μm) to obtain cell-free extracts with bioactive substances (EBS). The cell-free EBS were used directly (Table 2), and concentrations of 50 and 10% were obtained by dilution with Mueller-Hinton broth (MHB). Immediately, 200 μL of each concentration were transferred to 96-well microplates and inoculated with 10 μL of pathogenic bacteria (OD₆₂₀: 0.08). Three replicates and one contamination control were prepared and incubated at 37 °C for 24 h. Culture medium 2 × (TSB) supplemented with 0.2 or 0.02 mg/mL chloramphenicol served as the positive control (Valarezo et al., 2024). Growth of pathogenic bacteria was assessed using a magnifying glass; the minimum extract concentration that completely inhibited bacterial growth was considered the MIC, as per. Vega-portalatino et al. (2024). To determine the minimum bactericidal concentration (MBC), 100 μL from each well were subcultured onto Mueller-Hinton agar plates (without extract) and incubated at 37 °C for 24 h. The lowest concentration showing no bacterial growth was considered the MBC (Vega-portalatino et al., 2024).

**Table 2:** Concentrations of cell-free EBS (mg/mL) per selected bacterial culture.

ZA1	ZA5	ZA9	ZB3	ZB5	ZB7	ZAAgR1
42.95	43.99	47.44	45.16	44.62	44.07	44.62

ZA1: *Pseudomona monteilii*, ZA5 y ZA9: *Priestia flexa*, ZB3: *Bacillus albus,* ZB5: *Enterococcus faecalis*, ZB7: *Acinetobacter junii* y ZAAgR1*: Pseudomona monteilii*.

#### 2.3.6 Molecular identification of selected

The procedure was performed as described in Vega-portalatino et al. (2024). Seven bacteria from the MIC and MBC assays were cultured in Luria-Bertani broth (LB); pellets were recovered by centrifugation and DNA was extracted using the cetyltrimethylammonium bromide (CTAB) method. Primers 27F and 1492R were used for PCR amplification of 16S ribosomal DNA fragments. Agarose gel electrophoresis (1.5%) was used to assess amplicon quality. Sequencing of PCR products was performed using the Sanger method at Macrogen (Seoul, South Korea) with primers 518F and 800R. Sequence editing and assembly were performed using Chromas Lite and Cap3. Taxonomic group identification was performed based on reference sequences from GenBank using BLASTn.^1^ Phylogenetic trees were constructed using ClustalX v.2.1 for alignment and MEGA v.11 with the Neighbor-Joining algorithm, Kimura-2 parameter model, and 1,000 bootstrap replicates.

### 2.4 Biosafety assays

#### 2.4.1 Hemolytic activity

Two microliters of each bacterial suspension (OD₆₂₀: 0.08) were inoculated onto three sterile filter paper discs (6 mm), which were placed on plates containing tryptic soy agar (TSA) supplemented with 5% human blood and incubated at 30 °C for 48 h. Discs impregnated with 0.8% saline solution served as the negative control. The presence of green halos denotes alpha-hemolysis, yellow or clear halos denotes beta-hemolysis, and absence of a halo denotes gamma-hemolysis (Agurto-Fabiola et al., 2025).

#### 2.4.2 DNA degradation

Two microliters of each bacterial suspension (OD₆₂₀: 0.08) were inoculated onto three sterile filter paper discs (6 mm), which were placed on DNase agar plates and incubated at 30 °C for 48 h. Discs impregnated with 0.8% saline solution served as the negative control. The presence of green or clear halos is indicative of activity (Agurto-Fabiola et al., 2025). The hydrolysis zone was visualized using 1 N hydrochloric acid (HCl) (Fadare et al., 2023).

#### 2.4.3 Antibiotic susceptibility testing

Two standard discs of each of the following antimicrobial agents were used: Penicillin (10 μg), Tetracycline (30 μg), Rifampicin (5 μg), and Nalidixic acid (30 μg). The discs were placed on TSA plates previously inoculated by surface spreading with the bacterial suspensions (OD₆₂₀: 0.08). Plates were then incubated at 30 °C for 24 h under aerobic conditions. Inhibition halo diameters in millimeters (mm) were recorded and results were interpreted according to the guidelines of the Clinical and Laboratory Standards Institute (CLSI)(Valdiviezo-marcelo et al., 2023)(Agurto-Fabiola et al., 2025).

#### 2.4.4 Application of EBS from selected bacteria in the preservation of *Scomber japonicus peruanus* fillets

Strains showing the strongest responses in the MIC and MBC assays were selected. Fresh whole mackerel was purchased at the Mercado Modelo, Sullana, Piura, Peru, and washed with sterile distilled water according to the methodology of Morante-Carriel et al. (2023), then cut into fillet pieces (5 g) and placed in sterile Petri dishes. The pieces were immersed in 20 mL of EBS for 10 min, after which they were transferred to sterile Petri dishes sealed with Parafilm. Assays were conducted in duplicate; the negative control consisted of pieces immersed in TSB. The experiment comprised two incubation conditions:

- Ambient temperature (Ta): 27 ± 2 °C for 1 and 2 days.
- Refrigeration temperature (Tr): 7 ± 2 °C for 1 and 7 days.

The response variables evaluated were: aerobic mesophile count (log₁₀ CFU/g), lactic acid bacteria (LAB) count (log₁₀ CFU/g), pH, and acidity percentage. The main effects of each factor and all two-way and three-way interactions were evaluated. Exact *p*-values and coefficients of determination (R^2^) are reported in Table 3. Since the evaluation periods differed between storage conditions, temperature, and time, the data were standardized as time (T): T_initial_ and T_final_ for ambient temperature and refrigeration conditions, respectively, in order to allow valid and homogeneous statistical comparison.

**Table 3:** Microbiological counts, physicochemical parameters, and three-factor factorial ANOVA of *Scomber japonicus peruanus* fillets treated with cell-free extracts during storage.

Strain	Storage condition	Time	Mesophiles Log_10_ (CFU/g)	LAB Log_10_ (CFU/g)	pH	Acidity (%)
A. Experimental data						
ZA1	Refrigeration	t_initial (day 1)_	3.389 ± 0.013ᵃ	0.000 ± 0.000ᵃ	5.750 ± 0.071ᵃ	0.750 ± 0.071ᶜᵈ
		t_final (day 7)_	6.283 ± 0.013ᶠ	0.000 ± 0.000ᵃ	6.900 ± 0.000ᵉ	0.200 ± 0.000ᵉ
ZB3		t_initial (day 1)_	4.125 ± 0.004ᵇ	0.000 ± 0.000ᵃ	5.800 ± 0.141ᵃᵇ	0.850 ± 0.071ᶜ
		t_final (day 7)_	6.286 ± 0.003ᶠ	0.000 ± 0.000ᵃ	7.200 ± 0.000ᶠ	0.050 ± 0.000ᵉ
ZAAgR1		t_initial (day 1)_	4.121 ± 0.001ᵇ	0.000 ± 0.000ᵃ	5.750 ± 0.071ᵃ	1.150 ± 0.071ᵇ
		t_final (day 7)_	6.187 ± 0.024ᵉ	0.000 ± 0.000ᵃ	6.950 ± 0.071ᵉᶠ	0.100 ± 0.000ᵉ
C(−)		t_initial (day 1)_	4.091 ± 0.004ᵇ	0.000 ± 0.000ᵃ	5.750 ± 0.071ᵃ	0.600 ± 0.000ᶜᵈ
		t_final (day 7)_	6.362 ± 0.005ᵍ	0.000 ± 0.000ᵃ	7.200 ± 0.000ᶠ	0.010 ± 0.000ᵉ
ZA1	Ambient	t_initial (day 1)_	5.589 ± 0.013ᶜ	5.885 ± 0.035ᵉ	6.050 ± 0.071ᵇ	1.350 ± 0.071ᵃᵇ
		t_final (day 2)_	6.462 ± 0.030ʰ	6.405 ± 0.071^g^	6.850 ± 0.071ᵈᵉ	0.750 ± 0.212ᶜᵈ
ZB3		t_final (day 1)_	5.832 ± 0.009ᵈ	5.480 ± 0.141ᵈ	5.900 ± 0.000ᵃᵇ	1.200 ± 0.000ᵃᵇ
		t_final (day 2)_	6.158 ± 0.017ᵉ	6.090 ± 0.127ᵉᶠ	6.600 ± 0.141ᶜᵈ	0.650 ± 0.071ᶜᵈ
ZAAgR1		t_initial (day 1)_	5.544 ± 0.018ᶜ	4.900 ± 0.071ᵇ	5.950 ± 0.071ᵃᵇ	1.450 ± 0.071ᵃ
		t_final (day 2)_	6.606 ± 0.006ⁱ	6.115 ± 0.050ᶠ	6.850 ± 0.071ᵈᵉ	0.550 ± 0.071ᵈ
C(−)		t_initial (day 1)_	5.872 ± 0.021ᵈ	5.175 ± 0.035ᶜ	6.550 ± 0.071ᶜ	1.250 ± 0.071ᵃᵇ
		t_final (day 2)_	6.919 ± 0.007ʲ	6.255 ± 0.035ᶠᵍ	6.800 ± 0.000ᵈᵉ	1.300 ± 0.000ᵃᵇ
B. Factorial ANOVA—*p*-values						
Strain			0.000	0.000	0.019	0.000
Storage condition			0.000	0.243	0.000	0.000
Time			0.000	0.000	0.000	0.000
Strain × storage condition			0.000	0.000	0.000	0.000
Strain × time			0.000	0.045	0.000	0.000
Storage condition × time			0.000	0.000	0.000	0.000
Strain × storage condition × time			0.000	0.000	0.002	0.000
R^2^			99.99%	99.09%	98.84%	99.98%

ZA1: *Pseudomonas monteilii*; ZB3: *Bacillus albus*; ZAAgR1: *Pseudomonas monteilii*; C(−): negative control (TSB). Values in Block A represent the mean of two technical replicates ± SD. Different superscript letters within the same column indicate statistically significant differences according to Tukey’s post-hoc test (p < 0.05). T_initial_: Day 1 for both conditions; T_final_: Day 2 (ambient temperature, 27 ± 2°C) and Day 7 (refrigeration, 7 ± 2°C). Block B presents p-values from the three-factor factorial ANOVA processed with Minitab v.18. Italic value indicates the only non-significant main effect (*p* = 0.243). LAB: lactic acid bacteria. Detection limit: 1.0 log₁₀ CFU/g.

#### 2.4.5 Microbiological analysis

At each sampling time, mackerel pieces were analyzed using the serial dilution technique. For ambient temperature conditions, 100 μL of the 10^−1^, 10^−3^, and 10^−4^ dilutions were inoculated by surface spreading at 0, 24, and 48 h; for refrigeration conditions, 100 μL of the 10^−1^, 10^−2^, and 10^−5^ dilutions were spread at 0, 1, 4, and 7 days. TSA plates were used for the enumeration of aerobic mesophiles, and de Man, Rogosa, and Sharpe agar (MRS) supplemented with 1% CaCO₃ was used for lactic acid bacteria (LAB) enumeration. Both assays were incubated at 37 °C for 48 h and performed in duplicated. Fish immersed in TSB served as the negative control. The detection limit was 1.0 × 10^1^ CFU/g (1.0 log₁₀ CFU/g), calculated based on the minimum inoculum volume and the dilution factor employed.

#### 2.4.6 pH and acidity determination

Five grams of each fish piece were diluted in 50 mL of 0.8% NaCl, with three replicates per sample. pH was measured using a pH meter, and acidity was determined by volumetric titration using the following Equation 1, according to Agurto-Fabiola et al. (2025). Both parameters were evaluated at the end of each incubation period.


%Acidity=[(Vc×N×Ew)/(Sv×1000)]×100 (1)

Where:

Vc: Volume of NaOH used in the titration.
N: Normality of the standard NaOH solution.
Ew: Equivalent weight of lactic acid (90.08 g).
Sv: Sample volume.
1,000: Conversion factor (mg g^−1^).

### 2.5 Statistical analysis

All assays were performed with 2 or 3 replicates. The VOCt and OpT assays were analyzed using the mean and standard deviation (SD), analysis of variance (ANOVA), and Tukey’s test (*α* = 0.05) with the Statistical Package for the Social Sciences (SPSS), version 23. For the preservation of *Scomber japonicus peruanus* fillets, a three-factor factorial ANOVA (strain × storage condition × time) was applied, followed by Tukey’s post-hoc test (α = 0.05) using the statistical package MINITAB v18. The MIC and MBC assays were evaluated qualitatively.

## 3 Results

### 3.1 Sample collection and bacterial isolation

Twenty-one bacteria were isolated from sediment, of which 10 strains originated from Zone A and 11 from Zone B, coded as ZA and ZB, respectively. With respect to *Avicennia germinans* samples, 8 bacteria were isolated from roots, 4 from leaves, and 1 from the stem, as detailed in Table 4.

**Table 4:** Endophytic bacteria isolated from *Avicennia germinans* collected from the Vichayal Mangroves (Northern Peru, Piura).

Plant organ	Codes	Number of isolated bacteria	Isolation percentage (%)
Leaves	ZAAgH	4	30.8
Stems	ZAAgT	1	7.7
Roots	ZAAgR	8	61.5

Additionally, the physicochemical water parameters of Zone B were higher than those of Zone A with respect to T, EC, Ha, Alk, and Tu. However, pH and DO were higher in Zone A. No differences between zones were observed for Cl, as shown in Table 1.

### 3.2 Antimicrobial assays

#### 3.2.1 VOCt y OpT

**Figure 2:**
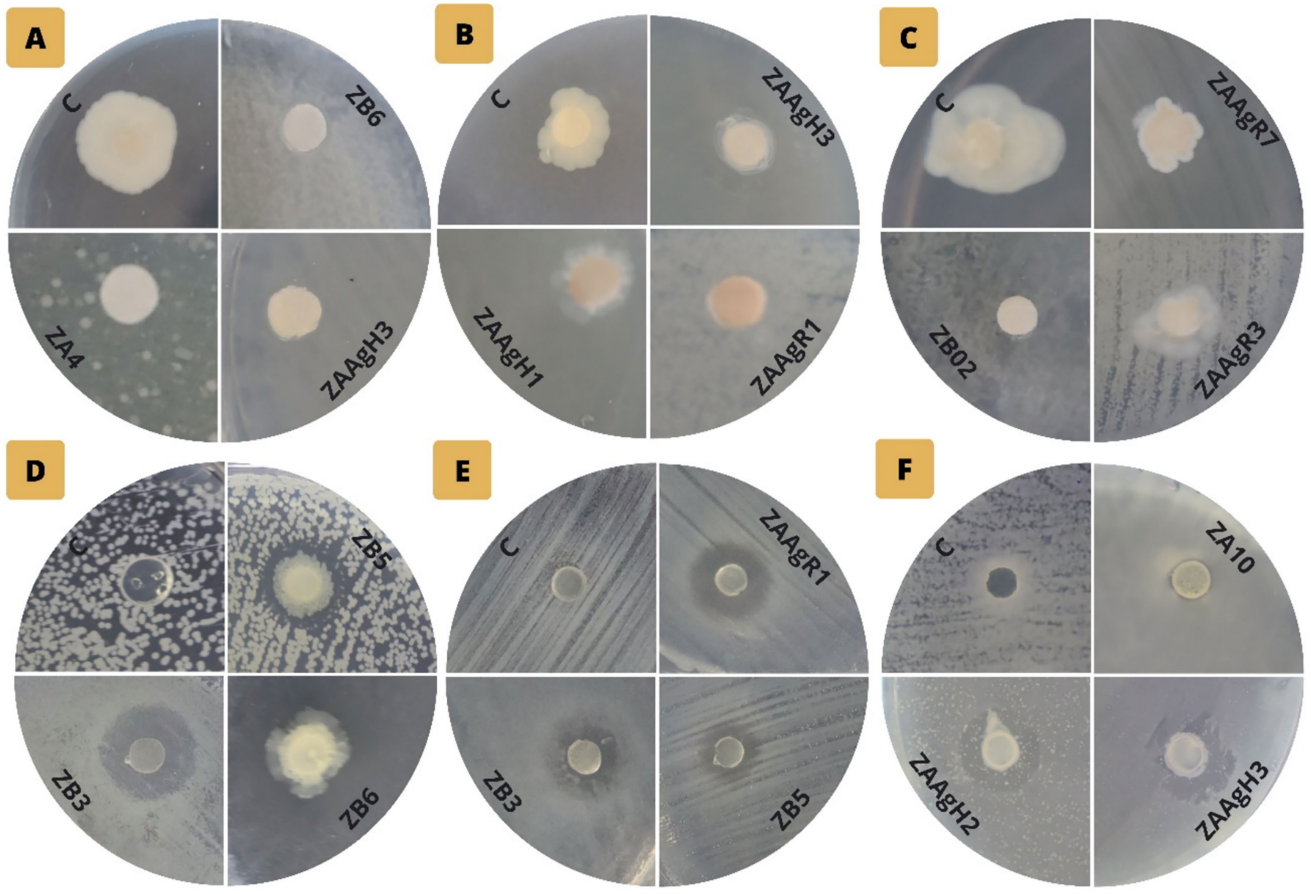
Antibacterial activity assessed by VOCt and OpT of bacteria isolated from sediment and *Avicennia germinans*. *VOCt assay*, **(A)** Inhibition of Gram-positive pathogenic bacteria (ZB6 inhibiting *Staphylococcus aureus* ATCC25923; ZAAgH3 inhibiting *Staphylococcus epidermidis* ATCC12228; ZA4 inhibiting *Listeria monocytogenes* ATCC7644); **(B)** Inhibition of Gram-negative pathogenic bacteria (ZAAgH3 inhibiting *Escherichia coli* ATCC10536; ZAAgR1 inhibiting *Escherichia coli* O157:H7; ZAAgH1 inhibiting *Salmonella enterica* sv. *typhimurium* ATCC14028); and **(C)** Inhibition of pathogenic yeasts (ZAAgR7 inhibiting *Candida albicans* ATCC90028; ZAAgR3 and ZB02 inhibiting *Candida tropicalis* ATCC750). *OpT assay*, **(D)** Inhibition of Gram-positive pathogenic bacteria (ZB5 inhibiting *Staphylococcus aureus* ATCC25923; ZB6 inhibiting *Enterococcus faecalis* ATCC29212; ZB3 inhibiting *Staphylococcus epidermidis* ATCC12228); **(E)** Inhibition of Gram-negative pathogenic bacteria (ZAAgR1 inhibiting *Escherichia coli* O157:H7; ZB5 inhibiting *Escherichia coli* ATCC10536; ZB3 inhibiting *Salmonella enterica* sv. *typhimurium* ATCC14028); and **(F)** Inhibition of pathogenic yeasts (ZA10 inhibiting *Candida albicans* ATCC90028; ZAAgH3 and ZAAgH2 inhibiting *Candida tropicalis* ATCC750). Growth control (C): without sediment or mangrove bacteria.

Of the 34 bacteria tested in the VOCt assay, 3, 4, 5, 7, 23, and 24 strains showed the greatest inhibition against *S. aureus* ATCC29213, *E. faecalis*, *S. aureus* ATCC700699, *L. monocytogenes*, *S. aureus* ATCC25923, and *S. epidermidis*, respectively. In the OpT assay, strains ZB3, ZB5, ZB3, and ZAAgR1 were able to inhibit *S. aureus* ATCC29213, *S. aureus* ATCC25923, *S. epidermidis*, and *E. faecalis*, respectively; however, none of the strains showed a response against *S. aureus* ATCC700699 or *L. monocytogenes* in the OpT assay, as shown in Table 5 and Figures 2A,D.

**Table 5:** Antibacterial activity of bacteria isolated from sediment and *Avicennia germinans* against Gram-positive pathogenic bacteria.

Strains	Sa13		Sa23		Sa99		Se		Ef		Lm		Activity threshold
	VOCt	OpT	VOCt	OpT	VOCt	OpT	VOCt	OpT	VOCt	OpT	VOCt	OpT	
ZA01	100 ± 0.0^a^	-	100 ± 0.0^a^	-	40 ± 0.0^d^	-	100 ± 0.0^a^	-	-	-	89 ± 0.0^b^	-	++
ZA02	40 ± 0.0^d^	-	93 ± 0.0^c^	-	40 ± 0.0^d^	-	100 ± 0.0^a^	-	75 ± 0.0^b^	-	78 ± 0.0^c^	-	+
ZA03	27 ± 4.6^e^	-	100 ± 0.0^a^	-	60 ± 0.0^c^	-	100 ± 0.0^a^	-	25 ± 0.0^d^	-	48 ± 6.4^f^	-	++
ZA04	60 ± 0.0^c^	-	100 ± 0.0^a^	-	100 ± 0.0^a^	-	75 ± 0.0^b^	-	75 ± 0.0^b^	-	100 ± 0.0^a^	-	++
ZA05	40 ± 0.0^d^	-	100 ± 0.0^a^	-	100 ± 0.0^a^	-	100 ± 0.0^a^	-	-	3.7 ± 0.6^e^	89 ± 0.0^b^	-	++
ZA06	40 ± 0.0^d^	-	95 ± 4.12^b^	-	20 ± 0.0^e^	-	100 ± 0.0^a^	-	50 ± 0.0^c^	-	100 ± 0.0^a^	-	++
ZA07	60 ± 0.0^c^	-	100 ± 0.0^a^	-	20 ± 0.0^e^	-	100 ± 0.0^a^	-	75 ± 0.0^b^	-	67 ± 0.0^d^	-	++
ZA08	20 ± 0.0^f^	-	93 ± 0.0^c^	-	40 ± 0.0^d^	-	100 ± 0.0^a^	-	-	-	100 ± 0.0^a^	-	++
ZA09	60 ± 0.0^c^	-	100 ± 0.0^a^	6.0 ± 0.0^b^	60 ± 0.0^c^	-	100 ± 0.0^a^	-	25 ± 0.0^d^	-	100 ± 0.0^a^	-	+++
ZA10	60 ± 0.0^c^	-	100 ± 0.0^a^	8.7 ± 0.6^b,c^	60 ± 0.0^c^	-	75 ± 0.0^b^	-	-	-	44 ± 0.0^g^	-	+
ZB01	100 ± 0.0^a^	-	79 ± 0.0^e^	-	40 ± 0.0^d^	-	100 ± 0.0^a^	-	33.3 ± 0.0^d^	-	89 ± 0.0^b^	-	+
ZB02	40 ± 0.0^d^	-	100 ± 0.0^a^	-	20 ± 0.0^e^	-	100 ± 0.0^a^	-	100 ± 0.0^a^	-	100 ± 0.0^a^	-	++
ZB03	20 ± 0.0^f^	10 ± 0.6^a^	100 ± 0.0^a^	5.0 ± 0.0^b^	80 ± 0.0^b^	-	75 ± 0.0^b^	15.3 ± 0.6^a^	75 ± 0.0^b^	-	78 ± 0.0^c^	-	+++
ZB04	60 ± 0.0^c^	-	93 ± 0.0^c^	-	100 ± 0.0^a^	-	50 ± 0.0^c^	-	-	-	56 ± 0.0^e^	-	+
ZB05	20 ± 0.0^f^	-	100 ± 0.0^a^	10 ± 0.0^a^	40 ± 0.0^d^	-	75 ± 0.0^b^	-	50 ± 0.0^c^	10.0 ± 0.0^b^	67 ± 0.0^d^	-	+++
ZB06	40 ± 0.0^d^	-	100 ± 0.0^a^	-	60 ± 0.0^c^	-	50 ± 0.0^c^	-	100 ± 0.0^a^	8.7 ± 0.6^c^	56 ± 0.0^e^	-	++
ZB07	100 ± 0.0^a^	-	100 ± 0.0^a^	-	80 ± 0.0^b^	-	42 ± 7.2^d^	-	-	-	78 ± 0.0^c^	-	++
ZB08	20 ± 0.0^f^	-	93 ± 0.0^c^	-	20 ± 0.0^e^	-	100 ± 0.0^a^	-	-	-	89 ± 0.0^b^	-	+
ZB09	80 ± 0.0^b^	-	100 ± 0.0^a^	-	20 ± 1.2^e^	-	100 ± 0.0^a^	-	75 ± 0.0^b^	-	67 ± 0.0^d^	-	++
ZB10	20 ± 0.0^f^	-	100 ± 0.0^a^	-	60 ± 0.0^c^	-	100 ± 0.0^a^	-	75 ± 0.0^b^	-	89 ± 0.0^b^	-	++
ZB11	-	-	100 ± 0.0^a^	-	80 ± 0.0^b^	-	100 ± 0.0^a^	-	25 ± 0.0^d^	-	100 ± 0.0^a^	-	++
ZAAgH1	40 ± 0.0^d^	-	100 ± 0.0^a^	-	100 ± 0.0^a^	-	100 ± 0.0^a^	-	25 ± 0.0^d^	-	89 ± 0.0^b^	-	++
ZAAgH2	80 ± 0.0^b^	-	86 ± 0.0^d^	-	80 ± 0.0^b^	-	100 ± 0.0^a^	-	50 ± 0.0^c^	-	89 ± 0.0^b^	-	+
ZAAgH3	20 ± 0.0^f^	-	93 ± 0.0^c^	-	100 ± 0.0^a^	-	100 ± 0.0^a^	-	100 ± 0.0^a^	-	100 ± 0.0^a^	-	++
ZAAgH4	-	-	93 ± 0.0^c^	-	80 ± 0.0^b^	-	100 ± 0.0^a^	-	-	-	89 ± 0.0^b^	-	+
ZAAgT1	40 ± 0.0^d^	-	93 ± 0.0^c^	-	40 ± 0.0^d^	-	75 ± 0.0^b^	-	-	-	89 ± 0.0^b^	-	+
ZAAgR1	20 ± 0.0^f^	-	100 ± 0.0^a^	-	40 ± 0.0^d^	-	100 ± 0.0^a^	-	75 ± 0.0^b^	11.0 ± 0.6^a^	78 ± 0.0^c^	-	+++
ZAAgR2	-	-	93 ± 0.0^c^	-	40 ± 0.0^d^	-	100 ± 0.0^a^	-	100 ± 0.0^a^	-	89 ± 0.0^b^	-	++
ZAAgR3	80 ± 0.0^b^	-	100 ± 0.0^a^	-	-	-	100 ± 0.0^a^	-	50 ± 0.0^c^	-	89 ± 0.0^b^	-	++
ZAAgR4	20 ± 0.0^f^	-	100 ± 0.0^a^	-	80 ± 0.0^b^	-	100 ± 0.0^a^	-	75 ± 0.0^b^	-	78 ± 0.0^c^	-	++
ZAAgR5	40 ± 0.0^d^	-	100 ± 0.0^a^	-	-	-	100 ± 0.0^a^	-	50 ± 0.0^c^	-	56 ± 0.0^e^	-	++
ZAAgR6	20 ± 0.0^f^	-	100 ± 0.0^a^	-	80 ± 0.0^b^	-	100 ± 0.0^a^	-	-	-	56 ± 0.0^e^	-	++
ZAAgR7	80 ± 0.0^b^	-	100 ± 0.0^a^	-	40 ± 0.0^d^	-	75 ± 0.0^b^	-	50 ± 0.0^c^	-	78 ± 0.0^c^	-	+
ZAAgR8	20 ± 0.0^f^	-	100 ± 0.0^a^	-	40 ± 0.0^d^	-	75 ± 0.0^b^	-	-	4.3 ± 0.6^d^	22 ± 0.0^h^	-	+

OpT: Over Plate Test (mm); VOCt: Volatile Organic Compounds Test (percentage inhibition). Sa13: *Staphylococcus aureus* ATCC29213; Sa23: *Staphylococcus aureus* ATCC25923; Sa99: *Staphylococcus aureus* ATCC700699; Se: *Staphylococcus epidermidis* ATCC12228; Ef: *Enterococcus faecalis* ATCC29212; Lm: *Listeria monocytogenes* ATCC7644. (−): no inhibitory activity. Values represent the mean of three replicates ± SD. Letters indicate groups with statistically significant differences according to Tukey’s test (*P* < 0.05). Inhibition threshold (VOCt: 100% and OpT: subscript a,b) relative to control: (+++) strong inhibition (≥ 2 strains and subscript a,b); (++) moderate inhibition (≥ 2 strains and no inhibitory activity); (+) weak inhibition (1 strain and no inhibitory activity).

In the VOCt assay, 14, 8, and 20 strains achieved strong inhibition against *E. coli* O157:H7, *E. coli* ATCC10536, and *S. enterica* sv. *typhimurium*, respectively. In the OpT assay, ZAAgR1, ZB05, ZA10, and ZB3 showed high inhibitory activity against *E. coli* O157:H7, *E. coli* ATCC10536, and *S. enterica* sv. *typhimurium*, as shown in Table 6 and Figures 2B,E.

**Table 6:** Antibacterial activity of bacteria isolated from sediment and *Avicennia germinans* against gram-negative pathogenic bacteria.

Strains	EcH7		Ec36		Sety		Activity threshold
	VOCt	OpT	VOCt	OpT	VOCt	OpT	
ZA01	75 ± 0.0^b^	−	100 ± 0.0^a^	−	89 ± 0.0^a,b,c,d^	−	++
ZA02	25 ± 0.0^e^	−	100 ± 0.0^a^	−	91 ± 3.0^a,b,c^	−	++
ZA03	75 ± 0.0^b^	−	75 ± 0.0^c^	−	93 ± 3.0^a,b^	−	+
ZA04	100 ± 0.0^a^	−	88 ± 0.0^b^	−	86 ± 3.0^b,c,d,e^	−	+
ZA05	100 ± 0.0^a^	3.7 ± 0.6^d^	63 ± 0.0^d^	−	87 ± 3.0^a,b,c,d,e^	−	++
ZA06	−	−	50 ± 0.0^e^	−	81 ± 3.0^d,e^	−	+
ZA07	75 ± 0.0^b^	−	88 ± 0.0^b^	−	93 ± 3.0^a,b^	−	+
ZA08	25 ± 0.0^e^	−	38 ± 0.0^g^	−	95 ± 3.0^a^	−	+
ZA09	100 ± 0.0^a^	−	63 ± 0.0^d^	5.3 ± 0.6^b^	79 ± 0.0^e^	−	++
ZA10	100 ± 0.0^a^	−	75 ± 0.0^c^	8.7 ± 0.2^a,b^	79 ± 0.0^e^	−	++
ZB01	33 ± 0.0^d^	−	88 ± 0.0^b^	−	89 ± 0.0^a,b,c,d^	−	+
ZB02	100 ± 0.0^a^	−	88 ± 0.0^b^	−	86 ± 3.0^b,c,d,e^	−	+
ZB03	100 ± 0.0^a^	−	88 ± 0.0^b^	5.7 ± 0.6^b^	91 ± 3.0^a,b,c^	14.3 ± 0.6^a^	+++
ZB04	100 ± 0.0^a^	−	88 ± 0.0^b^	−	79 ± 0.0^e^	−	+
ZB05	50 ± 0.0^c^	10 ± 0.0^b^	100 ± 0.0^a^	10.0 ± 0.0^a^	93 ± 3.0^a,b^	−	+++
ZB06	25 ± 0.0^e^	9.3 ± 0.6^c^	75 ± 0.0^c^	−	95 ± 0.0^a^	−	+
ZB07	100 ± 0.0^a^	−	100 ± 0.0^a^	−	93 ± 3.0^a,b^	−	++
ZB08	75 ± 0.0^b^	−	63 ± 0.0^d^	−	84 ± 0.0^c,d,e^	−	+
ZB09	100 ± 0.0^a^	−	63 ± 0.0^d^	−	95 ± 0.0^a^	−	+
ZB10	100 ± 0.0^a^	−	75 ± 0.0^c^	−	93 ± 3.0^a,b^	−	+
ZB11	75 ± 0.0^b^	−	75 ± 0.0^c^	−	95 ± 0.0^a^	−	+
ZAAgH1	100 ± 0.0^a^	−	75 ± 0.0^c^	−	89 ± 0.0^a,b,c,d^	−	+
ZAAgH2	50 ± 0.0^c^	−	100 ± 0.0^a^	−	95 ± 0.0^a^	−	+
ZAAgH3	75 ± 0.0^b^	−	75 ± 0.0^c^	−	91 ± 3.0^a,b,c^	−	+
ZAAgH4	100 ± 0.0^a^	−	100 ± 0.0^a^	−	87 ± 3.0^a,b,c,d,e^	−	+
ZAAgT1	100 ± 0.0^a^	−	63 ± 0.0^d^	−	87 ± 3.0^a,b,c,d,e^	−	+
ZAAgR1	100 ± 0.0^a^	11.3 ± 0.6^a^	100 ± 0.0^a^	−	93 ± 3.0^a,b^	−	+++
ZAAgR2	50 ± 0.0^c^	−	50 ± 0.0^e^	−	86 ± 3.0^b,c,d,e^	−	+
ZAAgR3	25 ± 0.0^e^	−	100 ± 0.0^a^	−	86 ± 3.0^b,c,d,e^	−	+
ZAAgR4	50 ± 0.0^c^	−	75 ± 0.0^c^	−	86 ± 3.0^b,c,d,e^	−	+
ZAAgR5	−	−	46 ± 7.2^f^	−	86 ± 3.0^b,c,d,e^	−	+
ZAAgR6	−	−	88 ± 0.0^b^	−	93 ± 3.0^a.b^	−	+
ZAAgR7	−	−	75 ± 0.0^c^	−	93 ± 3.0^a,b^	−	+
ZAAgR8	−	−	88 ± 0.0^b^	−	81 ± 3.0^d,e^	−	+

OpT: Over Plate Test (mm); VOCt: Volatile Organic Compounds Test (percentage inhibition). EcH7: *Escherichia coli* O157: H7; Ec36: *Escherichia coli* ATCC10536; Sety: *Salmonella enterica* sv. typhimurium ATCC14028. (−): no inhibitory activity. Values represent the mean of three replicates ± SD. Letters indicate groups with statistically significant differences according to Tukey’s test (*P* < 0.05). Inhibition threshold (VOCt: 100% and OpT: subscript a,b) relative to control: (+++) strong inhibition (≥ 2 strains and subscript a,b); (++) moderate inhibition (1 strain and subscript a,b); (+) weak inhibition (1 strain and no inhibitory activity).

With respect to the anti-Candida assay, the VOCt revealed that 3 and 3 strains achieved strong inhibition against *C. albicans* and *C. tropicalis*, respectively. In the OpT assay, strains ZA10 and ZAAgH3 were able to inhibit *C. albicans* and *C. tropicalis*, respectively, as shown in Table 7 and Figures 2C,F.

**Table 7:** Anti-Candida activity of bacteria isolated from sediment and *Avicennia germinans*.

Strains	Ca		Ct		Activity threshold
	VOCt	OpT	VOCt	OpT	
ZA01	20 ± 0.0^f^	−	25 ± 0.0^e^	−	+
ZA02	60 ± 0.0^c^	−	75 ± 0.0^b^	−	+
ZA03	100 ± 0.0^a^	−	50 ± 0.0^c^	−	++
ZA04	80 ± 0.0^b^	−	50 ± 0.0^c^	−	+
ZA05	60 ± 0.0^c^	−	25 ± 0.0^e^	−	+
ZA06	60 ± 0.0^c^	−	50 ± 0.0^c^	−	+
ZA07	40 ± 0.0^d^	−	50 ± 0.0^c^	−	+
ZA08	80 ± 0.0^b^	−	25 ± 0.0^e^	−	+
ZA09	40 ± 0.0^d^	−	100 ± 0.0^a^	−	++
ZA10	60 ± 0.0^c^	1.2 ± 0.2^a^	−	−	++
ZB01	40 ± 0.0^d^	−	100 ± 0.0^a^	−	++
ZB02	80 ± 0.0^b^	−	100 ± 0.0^a^	−	++
ZB03	60 ± 0.0^c^	−	75 ± 0.0^b^	−	+
ZB04	20 ± 0.0^f^	−	75 ± 0.0^b^	−	+
ZB05	20 ± 0.0^f^	−	50 ± 0.0^c^	−	+
ZB06	−	−	50 ± 0.0^c^	−	+
ZB07	80 ± 0.0^b^	−	50 ± 0.0^c^	−	+
ZB08	40 ± 0.0^d^	−	25 ± 0.0^e^	−	+
ZB09	−	−	33 ± 0.0^d^	−	+
ZB10	20 ± 0.0^f^	−	75 ± 0.0^b^	−	+
ZB11	60 ± 0.0^c^	−	50 ± 0.0^c^	−	+
ZAAgH1	100 ± 0.0^a^	−	50 ± 0.0^c^	5.3 ± 0.3^d^	++
ZAAgH2	−	−	−	7.3 ± 0.3^c^	+
ZAAgH3	60 ± 0.0^c^	−	50 ± 0.0^c^	10.3 ± 0.3^a^	+
ZAAgH4	20 ± 0.0^f^	−	75 ± 0.0^b^	2.3 ± 0.3^f^	+
ZAAgT1	27 ± 2.3^e^	−	25 ± 0.0^e^	−	+
ZAAgR1	40 ± 0.0^d^	−	−	8.3 ± 0.3^b^	+
ZAAgR2	80 ± 0.0^b^	−	25 ± 0.0^e^	3.7 ± 0.3^e^	+
ZAAgR3	100 ± 0.0^a^	−	50 ± 0.0^c^	−	+
ZAAgR4	20 ± 0.0^f^	−	25 ± 0.0^e^	−	+
ZAAgR5	40 ± 0.0^d^	−	50 ± 0.0^c^	−	+
ZAAgR6	20 ± 0.0^f^	−	75 ± 0.0^b^	−	+
ZAAgR7	60 ± 0.0^c^	−	75 ± 0.0^b^	−	+
ZAAgR8	20 ± 0.0^f^	−	50 ± 0.0^c^	−	+

OpT: Over Plate Test (mm); VOCt: Volatile Organic Compounds Test (percentage inhibition). Ca: *Candida albicans* ATCC90028; Ct: *Candida tropicalis* ATCC750. (−): no inhibitory activity. Values represent the mean of three replicates ± SD. Letters indicate groups with statistically significant differences according to Tukey’s test (*P* < 0.05). Inhibition threshold (VOCt: 100% and OpT: subscript a,b) relative to control: (++) moderate inhibition (1 strain or subscript a,b); (+) weak inhibition.

#### 3.2.2 MIC and MBC

**Figure 3:**
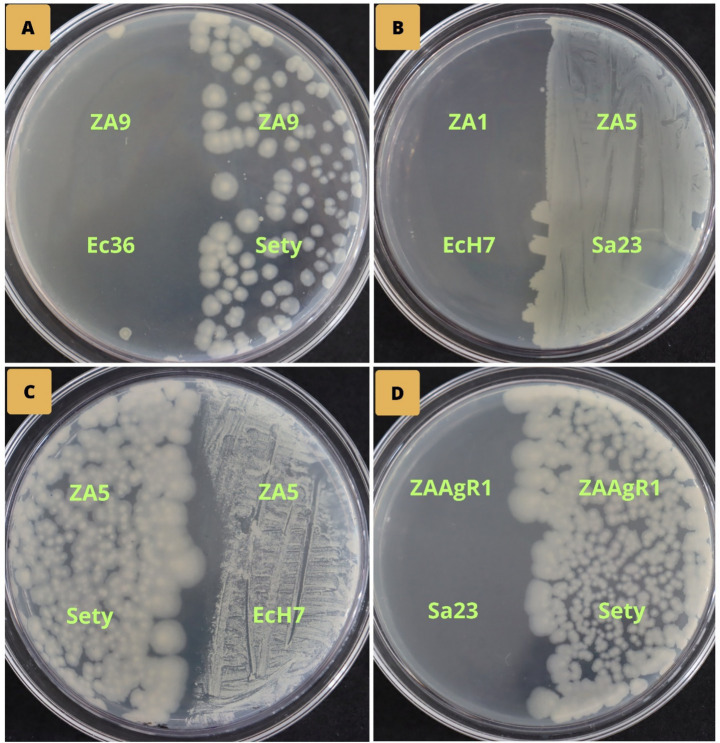
Antibacterial activity by MBC of bacteria isolated from sediment and *Avicennia germinans*. MBC assay, **(A)** 100% of extract ZA9 with effect on Ec36 and 100% of extract ZA9 without effect on Sety; **(B)** 10% of extract ZA1 with effect on EcH7 and 100% of extract ZA5 without effect on Sa23; **(C)** 100% of extract ZA5 without effect on Sety and EcH7; **(D)** 10% of extract ZAAgR1 with effect on Sa23 and without effect on Sety. Ec36: *Escherichia coli* ATCC10536; Sety: *Salmonella enterica* sv. *typhimurium* ATCC14028; EcH7: *Escherichia coli* O157:H7; Sa23: *Staphylococcus aureus* ATCC25923.

Seven bacterial strains were selected from the VOCt and OpT assays, based on strong and moderate inhibition thresholds against Gram-positive (Table 5) and Gram-negative (Table 6) pathogenic bacteria, to be tested against *S. aureus* ATCC25923, *S. epidermidis* ATCC12228, *E. coli* O157:H7, *E. coli* ATCC10536, and *S. enterica* sv. *typhimurium* ATCC14028. With respect to MIC, extracts from strains ZA1, ZA9, ZB3, ZB5, and ZAAgR1 (>10%) inhibited *S. aureus* ATCC25923 and *E. coli* O157:H7; additionally, bacteria ZA1, ZA5, ZB3, ZA9, and ZAAgR1 (100%) exerted inhibitory effects on at least 2 of the 3 additional pathogens tested (*S. epidermidis* ATCC12228, *E. coli* ATCC10536, and *S. enterica* sv. *typhimurium*). In the MBC assay, strains ZA1, ZB3, and ZAAgR1 (>10%) demonstrated activity against *S. aureus* ATCC25923 and *E. coli* O157:H7, while strains ZA1, ZA9, and ZAAgR1 (100%) successfully inhibited *E. coli* ATCC10536; however, no inhibitory response was observed against *S. epidermidis* ATCC12228 or *S. enterica* sv. *typhimurium*, as shown in Table 8 and Figures 3A–D.

**Table 8:** Antimicrobial activity of selected bacterial extracts against pathogenic bacteria by MIC and MBC, expressed as percentage.

Selected strains		Gram-positive pathogenic bacteria		Gram-negative pathogenic bacteria		
		Sa23	Se	EcH7	Ec36	Sety
ZA1	MIC	≥ 10%	+	≥ 10%	100%	100%
	MBC	≥ 10%	TN	≥ 10%	100%	+
ZA5	MIC	100%	100%	100%	+	100%
	MBC	+	+	+	TN	+
ZA9	MIC	≥ 10%	100%	≥ 10%	100%	100%
	MBC	+	+	+	100%	+
ZB3	MIC	≥ 10%	+	≥ 10%	100%	100%
	MBC	≥ 10%	TN	≥ 10%	+	+
ZB5	MIC	≥ 10%	+	≥ 10%	+	100%
	MBC	+	TN	+	TN	+
ZB7	MIC	+	+	+	+	+
	MBC	TN	TN	TN	TN	TN
ZAAgR1	MIC	≥ 10%	+	≥ 10%	100%	100%
	MBC	≥ 10%	TN	≥ 10%	100%	+
Chloramphenicol	mg/mL	0.02	0.2	0.02	0.02	0.02

Sa23: *Staphylococcus aureus* ATCC25923; Se: *Staphylococcus epidermidis* ATCC12228; EcH7: *Escherichia coli* O157: H7; Ec36: *Escherichia coli* ATCC10536; Sety: *Salmonella enterica* sv. typhimurium ATCC14028. (+): normal growth; NT: not tested.

#### 3.2.3 Phylogenetic analysis of selected strains

**Figure 4:**
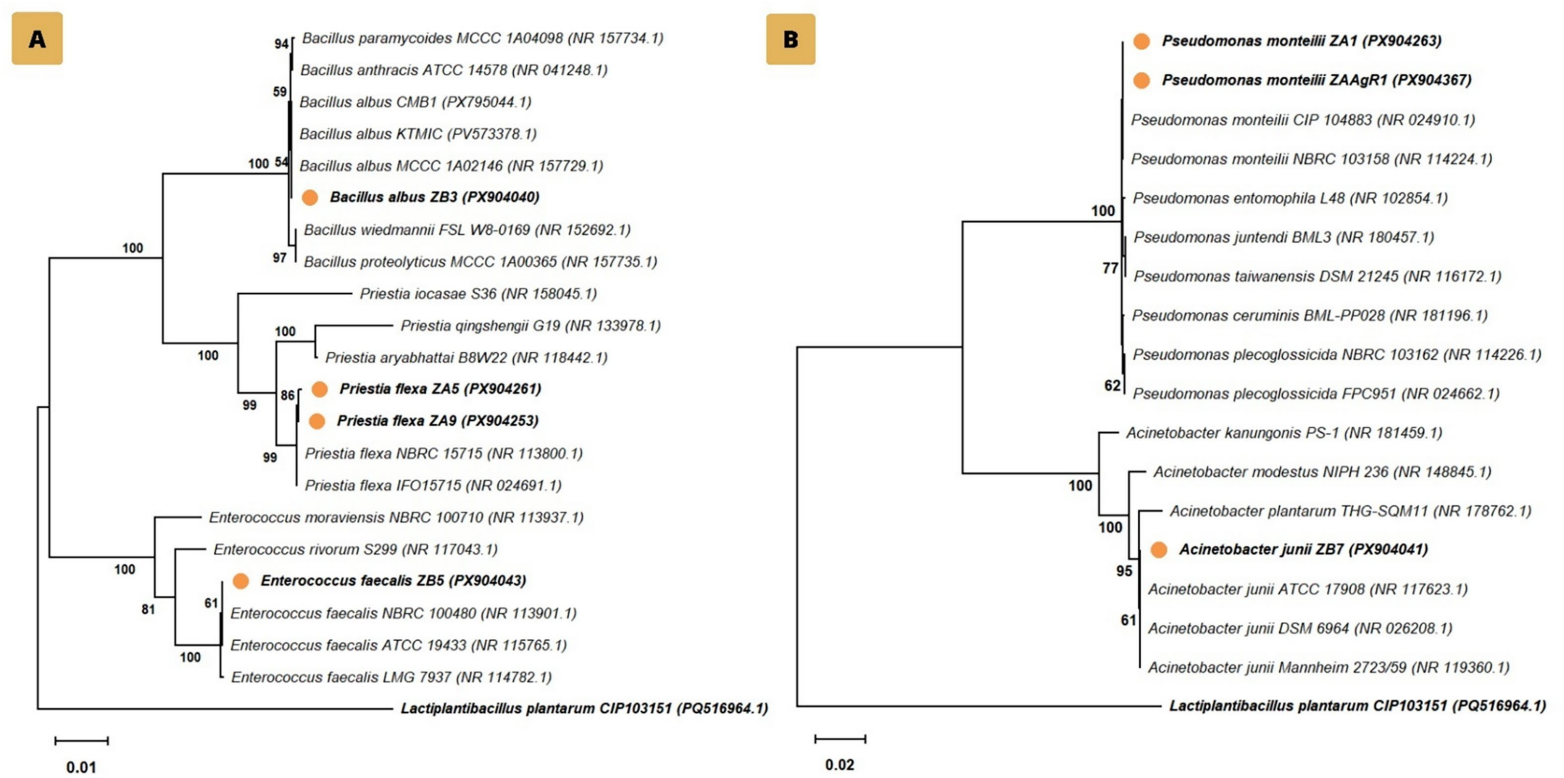
Phylogenetic analysis of selected bacteria from sediment and *Avicennia germinans* using 16S rDNA. **(A)** Group of Gram-positive bacteria and **(B)** Group of Gram-negative bacteria. Orange dots correspond to the isolated bacterial strains; remaining strains retrieved from GenBank are represented in non-bold italic type. The type strain is shown in bold italic. Accession numbers are presented in parentheses.

Of the seven previously selected bacteria, 4 Gram-positive bacteria were identified: *Bacillus albus* ZB3, *Priestia flexa* (ZA5 and ZA9), and *Enterococcus faecalis* ZB5. Additionally, 3 Gram-negative strains were identified: *Pseudomonas monteilii* (ZA1 and ZAAgR1) and *Acinetobacter junii* ZB7, as shown in Figures 4A,B.

#### 3.2.4 Biosafety assays

**Figure 5:**
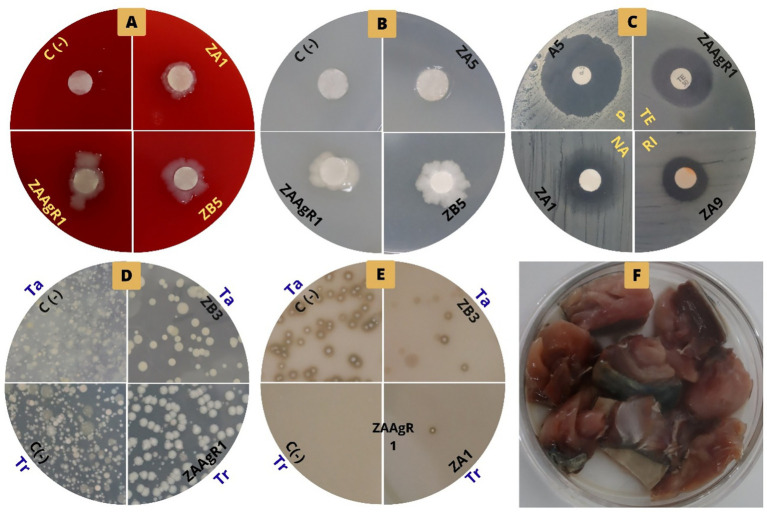
Biosafety assays of selected bacteria and evaluation of microbiological and physicochemical parameters in fish treated with extracts from selected bacteria isolated from sediment and *Avicennia germinans*. Biosafety assays: **(A)** Hemolytic activity, **(B)** DNA degradation, and **(C)** Antibiotic susceptibility (P: Penicillin; TE: Tetracycline; RI: Rifampicin; NA: Nalidixic acid). ZA1: *Pseudomonas monteilii*; ZA5 and ZA9: *Priestia flexa*; ZB3: *Bacillus albus*; ZB5: *Enterococcus faecalis*; ZB7: *Acinetobacter junii*; ZAAgR1: *Pseudomonas monteilii*. Fish piece preservation: **(D)** Aerobic mesophile count — ZB3 and ZAAgR1 with lower microbial load than the negative control; **(E)** Lactic acid bacteria count — ZB3 and ZA1 with lower microbial load than the negative control; **(F)** Fish samples treated with bacterial extracts. Values represent the mean of three replicates ± SD. NS: no significant differences. Letters indicate groups with statistically significant differences according to Tukey’s test (*p* < 0.05).

With respect to hemolytic activity, all 7 strains exhibited gamma-hemolysis (no activity). Regarding DNA degradation, all 7 strains did not produce DNase (Figures 5A–C). Finally, concerning antibiotic susceptibility, 85.7% of the strains were resistant to penicillin and tetracycline, and 100% were resistant to rifampicin and nalidixic acid (Table 9 and Figures 5A–C).

**Table 9:** Biosafety assays of selected bacteria.

Strains	Hemolytic activity	DNA degradation	Antibiotic sensitivity			
			P	TE	RI	NA
ZA1	γ	-	5 (R)	7 (R)	6 (R)	11 (R)
ZA5	γ	-	21 (S)	19 (I)	12 (R)	10 (R)
ZA9	γ	-	0 (R)	5 (R)	5 (R)	9 (R)
ZB3	γ	-	14 (R)	0 (R)	11 (R)	5 (R)
ZB5	γ	-	5 (R)	12 (R)	8 (R)	10 (R)
ZB7	γ	-	13 (R)	15 (I)	13 (R)	0 (R)
ZAAgR1	γ	-	2 (R)	11 (R)	8 (R)	10 (R)

ZA1: *Pseudomonas monteilii*; ZA5 and ZA9: Priestia flexa; ZB3: Bacillus albus; ZB5: *Enterococcus faecalis*; ZB7: *Acinetobacter junii*; ZAAgR1: *Pseudomonas monteilii*. (γ): gamma-hemolysis; (+): activity present; (−): no activity. P: Penicillin (10 μg); TE: Tetracycline (30 μg); RI: Rifampicin (5 μg); NA: Nalidixic acid (30 μg). R: Resistant (≤14 mm); I: Intermediate (15–19 mm); S: Susceptible (≥20 mm).

#### 3.2.5 Effect of cell-free EBS on the preservation of *Scomber japonicus peruanus*

The three-factor factorial ANOVA revealed that strain (*p* < 0.001), storage condition (p < 0.001), and time (p < 0.001) showed significant main effects on aerobic mesophile counts, lactic acid bacteria (LAB) counts, and acidity percentage (Table 3). For pH, storage condition did not show a significant independent main effect (*p* = 0.243), indicating that temperature alone did not determine pH variation, but rather its effect was mediated through interactions with the other factors. All two-way and three-way interactions were statistically significant (*p* < 0.05) for all response variables, confirming that the antimicrobial effect of each strain on microbial load and physicochemical parameters depended on both storage condition and evaluation time. Coefficients of determination exceeded 98% for all variables, confirming the high explanatory capacity of the factorial model applied.

With respect to aerobic mesophile counts, all treated fillets showed significantly lower values than the negative control at T-final under both storage conditions (*p* < 0.05). At ambient temperature (27 ± 2 °C), *Bacillus albus* ZB3 achieved the greatest reduction in mesophile counts (6.19 log₁₀ CFU/g) compared to the control (6.46 log₁₀ CFU/g) on day 2, while *Pseudomonas monteilii* ZAAgR1 showed the greatest LAB reduction (6.12 log₁₀ CFU/g) compared to the control (6.91 log₁₀ CFU/g). At refrigeration temperature (7 ± 2 °C), *P. monteilii* ZAAgR1 exhibited the lowest mesophile count on day 7 (6.36 log₁₀ CFU/g) relative to the control (6.28 log₁₀ CFU/g); no LAB growth was detected under refrigeration in any treatment, reflecting the inhibitory effect of low temperature on the proliferation of lactic acid bacteria, as shown in Figures 5D–F.

With respect to pH, values increased progressively from T-initial to T-final across all treatments and conditions, likely due to protein degradation and accumulation of metabolites during spoilage. At ambient temperature, the mean pH at T-final was 6.80, representing an increase of approximately 0.7 units relative to T-initial (6.09). At refrigeration temperature, pH values at T-final ranged between 6.90 (*B. albus* ZB3) and 7.20 (*P. monteilii* ZA1 and ZAAgR1), compared to the control (6.90). Regarding acidity, an initial increase was observed at T-initial under both conditions, followed by a reduction at T-final. At ambient temperature, *B. albus* ZB3 and *P. monteilii* ZAAgR1 reached the lowest acidity values on day 2 (0.55 and 0.55%, respectively), while under refrigeration all treatments showed acidity values close to 0.1% on day 7, possibly indicating metabolic stabilization of the fillet matrix during cold storage.

With respect to pH at ambient temperature, the mean pH value across strains was 6.8 ± 0.2 at 48 h of evaluation, representing an increase of 0.7 units compared to the initial pH (6.1 at time zero). At refrigeration temperature, strains *Pseudomonas monteilii* ZA1 and ZAAgR1 reached pH values of 6.9 and 7.0, respectively, compared to the control (7.2) evaluated at day seven, representing an increase of 0.8 units relative to the initial pH (6.1 at time zero). Regarding the percentage of acidity at ambient temperature, an increase was observed at 24 h (1.4 ± 0.2), followed by a reduction at 48 h, where strains *Pseudomonas monteilii* ZAAgR1 and *Bacillus albus* ZB3 reached a value of 0.7% compared to the control (1.3%). At refrigeration temperature, a slight increase was observed at day 1 (0.9 ± 0.3), with a reduction in acidity percentage by day seven across all evaluated bacteria (0.1 ± 0.1), as shown in Figures 5A–J.

## 4 Discussion

The diversity and composition of bacterial communities in mangrove forests play an important role within tropical and subtropical ecosystems, although they have not yet been fully explored (Di-Hua et al., 2022; Yuan et al., 2023). In the present study, 21 bacteria were isolated from sediment, with 52.4% originating from Zone A and 47.6% from Zone B; additionally, 13 endophytic bacteria were isolated, with the greatest number of isolates derived from the roots of *Avicennia germinans*. These results are consistent with the known bacterial abundance in the rhizosphere (Yuan et al., 2023; Lavanya et al., 2023; Wibowo et al., 2023), attributed to the direct contact roots maintain with nutrients, anaerobic/aerobic conditions, and diverse salinity and pH gradients, making them a favorable habitat for bacterial colonization (Mulyani et al., 2023; Azzahra et al., 2025), alongside sediment environments.

Physicochemical water parameters serve as quality indicators and play an important role in ecosystem equilibrium, exerting a direct influence on bacterial communities (Siriarchawatana et al., 2024; Arcela-castro et al., 2026). The physicochemical water parameters—T, pH, DO, EC, Ha, Alk, and Tu—varied between zones, with the exception of Cl. According to Azzahra et al. (2025) and Larekeng et al. (2026), fluctuations in temperature, pH, low oxygen levels, high salinity, and turbidity influence the adaptability and structure of mangrove-associated bacterial communities; for instance, ecosystems with elevated pH limit the biogeochemical metabolism of microbial communities, while salinity and turbidity confer greater protection against some natural inhibitors (Barik et al., 2018; Lai et al., 2022; Ceccon et al., 2019; George et al., 2021). Hardness is another important water quality parameter, though it is not a significant driver of bacterial community structure (Karaseva et al., 2024). Water alkalinity facilitates the displacement of proteins, improves the solubility of free amino acids, and allows the decomposition of protein nitrogen with the release of volatile nitrogenous compounds, conditions under which some bacteria proliferate more effectively while others are inhibited (Luo et al., 2025; Santhana lakshmi et al., 2023). The presence of chlorides affects the composition of bacterial communities, influencing microbial survival and behavior (Hassan et al., 2025; Sampaio et al., 2025).

Mangrove bacteria exhibit remarkable versatility in synthesizing a wide range of bioactive metabolites with diverse functionalities, including antimicrobials, offering broad industrial and biotechnological applications (Nor Hasan et al., 2024). Of the 34 bacteria from sediment and *Avicennia germinans*, 7 strains were selected based on the volatile organic compounds test (VOCt) and the disk diffusion test (OpT). The majority showed greater activity in the VOCt than in the OpT, suggesting that the antimicrobial activity is primarily associated with volatile compounds, a finding also confirmed by Vega-portalatino et al. (2024). Furthermore, VOCs are versatile agents against food pathogens as well as spoilage organisms; their application does not require direct contact with food, and represents an attractive strategy for controlling spoilage during storage and transport (Chandrasekaran et al., 2023; Zhao et al., 2022; Ma et al., 2022; Ulloa et al., 2023). In contrast, the OpT is related to the production of non-volatile compounds (Vega-portalatino et al., 2024), whose metabolites diffuse into the culture medium (Gajic et al., 2022).

In the MIC assay, 85.7% of the bacteria exhibited strong activity against *S. aureus* ATCC25923, *E. coli* O157: H7, and *S. enterica* sv. *typhimurium*, whereas the response against *S. epidermidis* ATCC12228 and *E. coli* ATCC10536 was lower. In the MBC assay, 28.6% of the strains demonstrated activity against *S. aureus* ATCC25923, *E. coli* O157:H7, and *E. coli* ATCC10536. These results are consistent with other reports on mangrove-associated strains with antimicrobial activity (Nor Hasan et al., 2024; Natania et al., 2024; Fatimah et al., 2022; Pringgenies and Setyati, 2023; Biswas et al., 2021), inhibition may be attributed to nutrient competition or antibiosis through the potential presence of bioactive substances such as terpenoids, alkaloids, peptides, polyketides, organic acids, or bacteriocins (Liu et al., 2026; Vega-portalatino et al., 2023; Lemos-lucumi et al., 2025; Wang and Fang, 2026), which would exert inhibitory effects on nucleic acid synthesis, energy metabolism, cell membrane functionality, or by suppressing cell wall synthesis (Biswas et al., 2021; Wang and Fang, 2026; Vega-portalatino et al., 2023).

Bacterial diversity was assessed using the 16S rDNA gene for molecular taxonomic identification (Vega-portalatino et al., 2024). The seven bacteria were identified as *Bacillus albus* ZB3, *Priestia flexa* (ZA5 and ZA9), *Enterococcus faecalis* ZB5, *Pseudomonas monteilii* (ZA1 and ZAAgR1), and *Acinetobacter junii* ZB7. *B. albus* is a species with demonstrated capacity to inhibit phytopathogenic microorganisms such as *Xanthomonas oryzae* (Chandwani et al., 2024), *Agrobacterium tumefaciens* (Yan et al., 2020), and *Aspergillus flavus* (Trinh et al., 2025), although no reports exist regarding its activity against pathogenic bacteria in food or clinical settings, nor are there studies linking it to mangrove environments. *P. flexa* has been previously reported from mangrove sediments in Central Java (Indonesia) (Setyati et al., 2021), the Vellar estuary in Parangipettai (Porto Novo, Tamil Nadu, India) (Chathalingath et al., 2023), Marine Drive in Cochin (Kerala, India) (Ruveena et al., 2025), as well as isolated from *Avicennia germinans* in Caribbean mangroves in Colombia (Soto-Varela et al., 2024) and from the Phra Chedi Klang Nam mangrove forests in Rayong, Thailand (Klinfoong et al., 2022), with demonstrated antimicrobial activity against *E. coli*, *S. aureus*, *L. monocytogenes*, and *Salmonella typhimurium* (Setyati et al., 2021; Kanzy et al., 2026) and *Bipolaris oryzae* (Paul and Chakraborty, 2025), and is valued in the industrial sector for its broad-spectrum antibiotic activity (Setyati et al., 2021). With respect to *E. faecalis*, this is a strain associated with human infections and a carrier of antibiotic resistance genes; its presence in mangrove sediments is attributed to anthropogenic activities such as inadequate wastewater disposal (Ibekwe et al., 2024; Santiago et al., 2024). This bacterium has also been shown to inhibit pathogenic bacteria (Yu et al., 2023), including *B. cereus*, *Klebsiella pneumoniae*, *E. coli*, *S. typhimurium*, and *Pseudomonas aeruginosa*, although few studies have reported it as a potential probiotic in food supplements (Tinrat et al., 2018).

*P. monteilii* is a species predominantly recognized as an environmental contaminant and opportunistic pathogen (Calhoun et al., 2025), isolated from mangrove sediments on Maparadita Island, Cartagena Bay, Colombia (Echeverri-Jaramillo et al., 2025), with beneficial antimicrobial properties against *Aeromonas hydrophila* (Qi et al., 2020) and high multidrug resistance; it has been used as a probiotic to control disease in fish (Qi et al., 2020), but is not considered safe as a food preservative for human use.

Finally, the genus *Acinetobacter* has been reported in other studies from mangrove forests collected in Ruwais and Al Thakhirah in Qatar (Yousefi et al., 2025), the Gaoqiao mangroves in Zhanjiang, Guangdong, China (Song et al., 2023), mangrove sediments from the Anil River in São Luís, Maranhão, Brazil (Santos Muniz et al., 2025), and the Sanya River riparian mangroves in southern Hainan, China (Huang et al., 2025). Although no reports exist for the species *A. junii* specifically associated with mangroves, this opportunistic strain has been reported with inhibitory capacity against *Proteus mirabilis*, *S. aureus*, and *P. aeruginosa* (Ohadi et al., 2020), though its use as a food preservative is not considered safe. In light of the foregoing, the strains selected in this study exhibit antimicrobial properties; however, genomic and biochemical characterization is necessary to elucidate the mechanistic basis of their activity.

The biosafety assays performed on the selected bacteria allow us to assess whether they are opportunistic pathogens (Agurto-Fabiola et al., 2025; Laborda et al., 2024). In this context, all 7 selected bacteria were found to exhibit gamma-hemolysis, indicating an absence of erythrocyte lysis and dissociating them from host tissue damage; furthermore, they did not synthesize DNase, confirming that the bacterial strains lack the capacity for nucleic acid degradation, thereby confirming their non-virulent nature and their inability to mount immune invasion (Borthakur et al., 2026; Alizadeh et al., 2024; Islam et al., 2026). Regarding antibiotic susceptibility, the majority exhibited resistance to penicillin, tetracycline, rifampicin, and nalidixic acid. Antibiotic resistance in the selected bacteria is a key parameter to be considered for safety assessment, which could favor fermentation and preservation processes when administered alongside antibiotics, and would not pose risks of horizontal transfer to non-pathogenic (Jeong et al., 2021; Agurto-Fabiola et al., 2025; Islam et al., 2023); however, they may represent opportunistic pathogens that would preclude their use in food preservation (Yi et al., 2025; Sharma and Mallubhotla, 2022).

With respect to the preservation of *Scomber japonicus peruanus*, at ambient temperature (27 ± 2 °C for 48 h), the extract of *Bacillus albus* ZB3 reduced aerobic mesophile and LAB counts; pH showed a slight increase of 8.1%, while acidity increased initially and subsequently returned to the initial value (0.7%). At refrigeration temperature (7 ± 2 °C for 7 days), the extract of *Pseudomonas monteilii* ZAAgR1 reduced aerobic mesophile counts; no LAB growth was observed. pH showed a slight increase (24 h) of 14.7%, while acidity increased initially and subsequently decreased to 0.1%. These findings are supported by Zawani et al. (2022), who reported that temperature and pH influence meat microbial counts, while treatment with microbial extracts retards the increase in total viable counts compared to the control, due to the presence of metabolites such as bacteriocins exerting inhibitory action. Accordingly, microbial growth is the most critical factor in fresh meat spoilage, causing the degradation of valuable proteins through amino acid catabolism, with accumulation of ammonia and decomposition products such as tyramine, putrescine, histamine, and cadaverine, leading to increased alkalinity, rancidity, and color changes (Zawani et al., 2022; Shange et al., 2019; Hussain et al., 2021; Neira Mosquera et al., 2026; Sobhan et al., 2022; Jiménez-Ruíz et al., 2023). The results of this study may contribute to the development of microbial agents capable of eliminating spoilage processes that threaten human health, to be used as preservatives to enhance product safety in the food industry and to inhibit pathogenic microorganisms of public health relevance (Pringgenies and Setyati, 2023).

## 5 Conclusion

A total of 34 bacteria were isolated from sediments (n = 21) and endophytic tissues of *Avicennia germinans* (n = 13) collected from the Vichayal mangroves (Piura, northern Peru), confirming that the rhizosphere and sediments are the most prolific sources of bacterial isolates within this ecosystem. Analysis by VOCt and OpT demonstrated broad-spectrum antimicrobial activity across the entire collection, with volatile compound-mediated inhibition predominating over the activity of diffusible metabolites. Seven strains—*Bacillus albus* ZB3, *Priestia flexa* (ZA5 and ZA9), *Enterococcus faecalis* ZB5, *Pseudomonas monteilii* (ZA1 and ZAAgR1), and *Acinetobacter junii* ZB7—identified by 16S rRNA gene sequencing exhibited the highest inhibitory performance, and strains ZA1, ZB3, and ZAAgR1 demonstrated the most consistent MIC and MBC activity against *S. aureus* ATCC25923, *E. coli* O157:H7, and *E. coli* ATCC10536 at extract concentrations ≥10%. Biosafety evaluation confirmed a non-virulent phenotype for all selected strains, as evidenced by gamma-hemolysis and absence of DNase activity; however, widespread resistance to clinically relevant antibiotics warrants caution regarding their direct application in food. Application of cell-free extracts with bioactive substances to *S. japonicus peruanus* fillets produced statistically significant reductions in aerobic mesophile and lactic acid bacteria counts under both ambient and refrigeration conditions, with concomitant modulation of pH and acidity, as confirmed by three-factor factorial ANOVA (R^2^ ≥ 98.84%). Collectively, these results indicate that *B. albus* ZB3 and *P. monteilii* ZAAgR1 represent the most promising candidates for the development of biopreservation strategies in fishery products; however, comprehensive genomic characterization and *in vivo* safety evaluation are required prior to their application in food matrices.

## Data Availability

The datasets presented in this study can be found in online repositories. The names of the repository/repositories and accession number(s) can be found in the article/Supplementary material.

## Footnotes

1
https://blast.ncbi.nlm.nih.gov/
