# Supplementary material for: Antimicrobial potential of bacteria isolated from sediment and Avicennia germinans collected from the mangroves of Vichayal (Northern Peru, Piura) and their application in the preservation of Scomber japonicus peruanus
Source: Front Microbiol. 2026 Jun 24;17:1879875. doi: 10.3389/fmicb.2026.1879875 (PMC13341936; doi:10.3389/fmicb.2026.1879875)
Supplement: Supplementary file 1 [file Supplementary_file_1.pdf]

SUPPLEMENTARY MATERIAL

Table 1. Close reference sequences of bacteria from sediment and *Avicennia germinans* obtained from GenBank.

Strain	Code	Number of base pairs (bp)	Accession number	Percentage identity	Query coverage	Bootstrap support values
Gram-positive bacteria						
*Priestia flexa**	ZA5	1202	PX904261	-	-	86
*Priestia flexa**	ZA9	1211	PX904253	-	-	86
*Bacillus albus**	ZB3	1204	PX904040	-	-	54
*Enterococcus faecalis**	ZB5	1196	PX904043	-	-	61
Priestia flexa	NBRC 15715	1478	NR 113800.1	99.83%	100%	99
Priestia_flexa	IFO15715	1529	NR 024691.1	99.83%	100%	99
Priestia qingshengii	G19	1455	NR 133978.1	97.33%	100%	100
Priestia aryabhattai	B8W22	1291	NR 118442.1	98.68%	95%	100
Priestia iocasae	S36	1510	NR 158045.1	96.34%	100%	100
Bacillus albus	MCCC 1A02146	1509	NR 157729.1	100%	100%	59
Bacillus albus	CMB1	1393	PX795044.1	100%	100%	59
Bacillus albus	KTMIC	1437	PV573378.1	100%	100%	59
Bacillus paramycoides	MCCC 1A04098	1509	NR 157734.1	99.92%	100%	94
Bacillus anthracis	ATCC 14578	1306	NR 041248.1	99.92%	98%	94
Bacillus wiedmannii	FSL W8-0169	1540	NR_152692.1	99.83%	100%	97
Bacillus proteolyticus	MCCC 1A00365	1509	NR 157735.1	99.83%	100%	97
Enterococcus faecalis	NBRC 100480	1426	NR 113901.1	100%	100%	61
Enterococcus faecalis	ATCC 19433	1483	NR 115765.1	100%	100%	61
Enterococcus faecalis	LMG 7937	1556	NR 114782.1	99.75%	100%	100
Enterococcus rivorum	S299	1479	NR 117043.1	98.49%	100%	81
Enterococcus moraviensis	NBRC 100710	1486	NR 113937.1	97.66%	100%	100
Gram-negative bacteria						
*Pseudomonas monteilii**	ZA1	1174	PX904263	-	-	100
*Pseudomonas monteilii**	ZAAgR1	1193	PX904041	-	-	61
*Acinetobacter junii**	ZB7	1225	PX904367	-	-	100
Pseudomonas monteilii	CIP 104883	1517	NR 024910.1	100%	100%	100

Pseudomonas monteilii	NBRC 103158	1462	NR_114224.1	99.91%	100%	100
Pseudomonas ceruminis	BML-PP028	1533	NR 181196.1	99.91%	100%	62
Pseudomonas juntendi	BML3	1432	NR 180457.1	99.83%	100%	77
Pseudomonas entomophila	L48	1526	NR 102854.1	99.83%	100%	77
Pseudomonas plecoglossicida	NBRC 103162	1462	NR 114226.1	99.83%	100%	62
Pseudomonas taiwanensis	DSM 21245	1469	NR 116172.1	99.83%	100%	77
Pseudomonas plecoglossicida	FPC951	1498	NR 024662.1	99.83%	100%	62
Acinetobacter junii	ATCC 17908	1529	NR 117623.1	100%	100%	61
Acinetobacter junii	DSM 6964	1459	NR 026208.1	100%	100%	61
Acinetobacter junii Mannheim	2723/59	1416	NR 119360.1	99.92%	100%	61
Acinetobacter plantarum	THG-SQM11	1452	NR 178762.1	98.79%	100%	95
Acinetobacter modestus	NIPH 236	1489	NR 148845.1	98.78%	100%	100
Acinetobacter_kanungonis	PS-1	1532	NR_181459.1	97.80%	100%	100
Lactiplantibacillus plantarum	CIP103151	1438	PQ516964.1	-	-	-

*Scientific names marked with an asterisk represent the strains selected from the antimicrobial assays.

Nucleotide sequences

>Priestia_flexa_ZA5_(PX904261)

ATACATGCAAGTCGAGCGAACTGATTAGAAGCTTGCTTCTATGACGTTAGCGGCGGACGGGTGAGTAACACGTGGGCAACCTGCCTGT

AAGACTGGGATAACTCCGGGAAACCGGAGCTAATACCGGATAACATTTTCTCTTGCATAAGAGAAAATTGAAAGATGGTTTCGGCTAT

CACTTACAGATGGGCCCGCGGTGCATTAGCTAGTTGGTGAGGTAACGGCTCACCAAGGCAACGATGCATAGCCGACCTGAGAGGGTGA

TCGGCCACACTGGGACTGAGACACGGCCCAGACTCCTACGGGAGGCAGCAGTAGGGAATCTTCCGCAATGGACGAAAGTCTGACGGA

GCAACGCCGCGTGAGTGATGAAGGCTTTCGGGTCGTAAAACTCTGTTGTTAGGGAAGAACAAGTACAAGAGTAACTGCTTGTACCTTG

ACGGTACCTAACCAGAAAGCCACGGCTAACTACGTGCCAGCAGCCGCGGTAATACGTAGGTGGCAAGCGTTATCCGGAATTATTGGGC

GTAAAGCGCGCGCAGGCGGTTTCTTAAGTCTGATGTGAAAGCCCACGGCTCAACCGTGGAGGGTCATTGGAAACTGGGGAACTTGAGT

GCAGAGAGAACAGCGGAATTCCACGTGTAGCGGTGAAATGCGTAGAGATGTGGAGGAACACCAGTGGCGAAGGCGGCTTTTTGGTCT

GTAACTGACGCTGAGGCGCGAAAGCGTGGGGAGCAAACAGGATTAGATACCCTGGTAGTCCACGCCGTAAACGATGAGTGCTAAGTG

TTAGAGGGTTTCCGCCCTTTAGTGCTGCAGCTAACGCATTAAGCACTCCGCCTGGGGAGTACGGTCGCAAGACTGAAACTCAAAGGAA

TTGACGGGGGCCCGCACAAGCGGTGGAGCATGTGGTTTAATTCGAAGCAACGCGAAGAACCTTACCAGGTCTTGACATCCTCTGACAA

CTCTAGAGATAGAGCGTTCCCCTTCGGGGGACAGAGTGACAGGTGGTGCATGGTTGTCGTCAGCTCGTGTCGTGAGATGTTGGGTTAAG

TCCCGCAACGAGCGCAACCCTTGATCTTAGTTGCCAGCATTTAGTTGGGCACTCTAAGGTGACTGCCGGTGACAAACCGGAGGAAGGT

GGGGATGACGTCAAATCATCATGCCCCTTATGACCTGGGCTACACACGTGCTACAATGGA

>Priestia_flexa_ZA9_(PX904253)

CGTGCCTAATACATGCAAGTCGAGCGAACTGATTAGAAGCTTGCTTCTATGACGTTAGCGGCGGACGGGTGAGTAACACGTGGGCAAC

CTGCCTGTAAGACTGGGATAACTCCGGGAAACCGGAGCTAATACCGGATAACATTTTCTCTTGCATAAGAGAAAATTGAAAGATGGTT

TCGGCTATCACTTACAGATGGGCCCGCGGTGCATTAGCTAGTTGGTGAGGTAACGGCTCACCAAGGCAACGATGCATAGCCGACCTGAGAGGG
TGATCGGCCACACTGGGACTGAGACACGGCCCAGACTCCTACGGGAGGCAGCAGTAGGGAATCTTCCGCAATGGACGAAAGT

CTGACGGAGCAACGCCGCGTGAGTGATGAAGGCTTTCGGGTCGTAAAACTCTGTTGTTAGGGAAGAACAAGTACAAGAGTAACTGCTT

GTACCTTGACGGTACCTAACCAGAAAGCCACGGCTAACTACGTGCCAGCAGCCGCGGTAATACGTAGGTGGCAAGCGTTATCCGGAAT

TATTGGGCGTAAAGCGCGCGCAGGCGGTTTCTTAAGTCTGATGTGAAAGCCCACGGCTCAACCGTGGAGGGTCATTGGAAACTGGGGA

ACTTGAGTGCAGAGAGAAAGCGGAATTCCACGTGTAGCGGTGAAATGCGTAGAGATGTGGAGGAACACCAGTGGCGAAGGCGGCTTT

TTGGTCTGTAACTGACGCTGAGGCGCGAAAGCGTGGGGAGCAAACAGGATTAGATACCCTGGTAGTCCACGCCGTAAACGATGAGTGC

TAAGTGTTAGAGGGTTTCCGCCCTTTAGTGCTGCAGCTAACGCATTAAGCACTCCGCCTGGGGAGTACGGTCGCAAGACTGAAACTCAA

AGGAATTGACGGGGGCCCGCACAAGCGGTGGAGCATGTGGTTTAATTCGAAGCAACGCGAAGAACCTTACCAGGTCTTGACATCCTCT

GACAACTCTAGAGATAGAGCGTTCCCCTTCGGGGGACAGAGTGACAGGTGGTGCATGGTTGTCGTCAGCTCGTGTCGTGAGATGTTGG

GTTAAGTCCCGCAACGAGCGCAACCCTTGATCTTAGTTGCCAGCATTTAGTTGGGCACTCTAAGGTGACTGCCGGTGACAAACCGGAG

GAAGGTGGGGATGACGTCAAATCATCATGCCCCTTATGACCTGGGCTACACACGTGCTACAATGGATG

>Bacillus_albus_ZB3_(PX904040)

TGCAAGTCGAGCGAATGGATTAAGAGCTTGCTCTTATGAAGTTAGCGGCGGACGGGTGAGTAACACGTGGGTAACCTGCCCATAAGAC

TGGGATAACTCCGGGAAACCGGGGCTAATACCGGATAACATTTTGAACCGCATGGTTCGAAATTGAAAGGCGGCTTCGGCTGTCACTT

ATGGATGGACCCGCGTCGCATTAGCTAGTTGGTGAGGTAACGGCTCACCAAGGCAACGATGCGTAGCCGACCTGAGAGGGTGATCGGC

CACACTGGGACTGAGACACGGCCCAGACTCCTACGGGAGGCAGCAGTAGGGAATCTTCCGCAATGGACGAAAGTCTGACGGAGCAAC

GCCGCGTGAGTGATGAAGGCTTTCGGGTCGTAAAACTCTGTTGTTAGGGAAGAACAAGTGCTAGTTGAATAAGCTGGCACCTTGACGG

TACCTAACCAGAAAGCCACGGCTAACTACGTGCCAGCAGCCGCGGTAATACGTAGGTGGCAAGCGTTATCCGGAATTATTGGGCGTAA

AGCGCGCGCAGGTGGTTTCTTAAGTCTGATGTGAAAGCCCACGGCTCAACCGTGGAGGGTCATTGGAAACTGGGAGACTTGAGTGCAG

AAGAGGAAAGTGGAATTCCATGTGTAGCGGTGAAATGCGTAGAGATATGGAGGAACACCAGTGGCGAAGGCGACTTTCTGGTCTGTA

ACTGACACTGAGGCGCGAAAGCGTGGGGAGCAAACAGGATTAGATACCCTGGTAGTCCACGCCGTAAACGATGAGTGCTAAGTGTTA

GAGGGTTTCCGCCCTTTAGTGCTGAAGTTAACGCATTAAGCACTCCGCCTGGGGAGTACGGCCGCAAGGCTGAAACTCAAAGGAATTG

ACGGGGGCCCGCACAAGCGGTGGAGCATGTGGTTTAATTCGAAGCAACGCGAAGAACCTTACCAGGTCTTGACATCCTCTGACAACCC

TAGAGATAGGGCTTCTCCTTCGGGAGCAGAGTGACAGGTGGTGCATGGTTGTCGTCAGCTCGTGTCGTGAGATGTTGGGTTAAGTCCCG

CAACGAGCGCAACCCTTGATCTTAGTTGCCATCATTAAGTTGGGCACTCTAAGGTGACTGCCGGTGACAAACCGGAGGAAGGTGGGGA

TGACGTCAAATCATCATGCCCCTTATGACCTGGGCTACACACGTGCTACAATGGACGGTACA

>Enterococcus_faecalis_ZB5_(PX904043)

CCGAGTGCTTGCACTCAATTGGAAAGAGGAGTGGCGGACGGGTGAGTAACACGTGGGTAACCTACCCATCAGAGGGGGATAACACTT

GGAAACAGGTGCTAATACCGCATAACAGTTTATGCCGCATGGCATAAGAGTGAAAGGCGCTTTCGGGTGTCGCTGATGGATGGACCCG

CGGTGCATTAGCTAGTTGGTGAGGTAACGGCTCACCAAGGCCACGATGCATAGCCGACCTGAGAGGGTGATCGGCCACACTGGGACTG

AGACACGGCCCAGACTCCTACGGGAGGCAGCAGTAGGGAATCTTCGGCAATGGACGAAAGTCTGACCGAGCAACGCCGCGTGAGTGA

AGAAGGTTTTCGGATCGTAAAACTCTGTTGTTAGAGAAGAACAAGGACGTTAGTAACTGAACGTCCCCTGACGGTATCTAACCAGAAA

GCCACGGCTAACTACGTGCCAGCAGCCGCGGTAATACGTAGGTGGCAAGCGTTGTCCGGATTTATTGGGCGTAAAGCGAGCGCAGGCG

GTTTCTTAAGTCTGATGTGAAAGCCCCCGGCTCAACCGGGGAGGGTCATTGGAAACTGGGAGACTTGAGTGCAGAAGAGGAGAGTGGA

ATTCCATGTGTAGCGGTGAAATGCGTAGATATATGGAGGAACACCAGTGGCGAAGGCGGCTCTCTGGTCTGTAACTGACGCTGAGGCT

CGAAAGCGTGGGGAGCAAACAGGATTAGATACCCTGGTAGTCCACGCCGTAAACGATGAGTGCTAAGTGTTGGAGGGTTTCCGCCCTT

CAGTGCTGCAGCAAACGCATTAAGCACTCCGCCTGGGGAGTACGACCGCAAGGTTGAAACTCAAAGGAATTGACGGGGGCCCGCACA

AGCGGTGGAGCATGTGGTTTAATTCGAAGCAACGCGAAGAACCTTACCAGGTCTTGACATCCTTTGACCACTCTAGAGATAGAGCTTTC

CCTTCGGGGACAAAGTGACAGGTGGTGCATGGTTGTCGTCAGCTCGTGTCGTGAGATGTTGGGTTAAGTCCCGCAACGAGCGCAACCC

TTATTGTTAGTTGCCATCATTTAGTTGGGCACTCTAGCGAGACTGCCGGTGACAAACCGGAGGAAGGTGGGGATGACGTCAAATCATC

ATGCCCCTTATGACCTGGGCTACACACGTGCTACAATGGGAAGTACAACGAGTC

>Pseudomonas_monteilii_ZA1_(PX904263)

TGCAAGTCGAGCGGATGACGGGAGCTTGCTCCTTGATTCAGCGGCGGACGGGTGAGTAATGCCTAGGAATCTGCCTGGTAGTGGGGGA

CAACGTTTCGAAAGGAACGCTAATACCGCATACGTCCTACGGGAGAAAGCAGGGGACCTTCGGGCCTTGCGCTATCAGATGAGCCTAG

GTCGGATTAGCTAGTTGGTGGGGTAATGGCTCACCAAGGCGACGATCCGTAACTGGTCTGAGAGGATGATCAGTCACACTGGAACTGA

GACACGGTCCAGACTCCTACGGGAGGCAGCAGTGGGGAATATTGGACAATGGGCGAAAGCCTGATCCAGCCATGCCGCGTGTGTGAA

GAAGGTCTTCGGATTGTAAAGCACTTTAAGTTGGGAGGAAGGGCAGTAAGTTAATACCTTGCTGTTTTGACGTTACCGACAGAATAAG

CACCGGCTAACTCTGTGCCAGCAGCCGCGGTAATACAGAGGGTGCAAGCGTTAATCGGAATTACTGGGCGTAAAGCGCGCGTAGGTGG

TTCGTTAAGTTGGATGTGAAAGCCCCGGGCTCAACCTGGGAACTGCATCCAAAACTGGCGAGCTAGAGTACGGTAGAGGGTGGTGGAA

TTTCCTGTGTAGCGGTGAAATGCGTAGATATAGGAAGGAACACCAGTGGCGAAGGCGACCACCTGGACTGATACTGACACTGAGGTGC

GAAAGCGTGGGGAGCAAACAGGATTAGATACCCTGGTAGTCCACGCCGTAAACGATGTCAACTAGCCGTTGGAATCCTTGAGATTTTA

GTGGCGCAGCTAACGCATTAAGTTGACCGCCTGGGGAGTACGGCCGCAAGGTTAAAACTCAAATGAATTGACGGGGGCCCGCACAAG

CGGTGGAGCATGTGGTTTAATTCGAAGCAACGCGAAGAACCTTACCAGGCCTTGACATGCAGAGAACTTTCCAGAGATGGATTGGTGC

CTTCGGGAACTCTGACACAGGTGCTGCATGGCTGTCGTCAGCTCGTGTCGTGAGATGTTGGGTTAAGTCCCGTAACGAGCGCAACCCTT

GTCCTTAGTTACCAGCACGTAATGGTGGGCACTCTAAGGAGACTGCCGGTGACAAACCGGAGGAAGGTGGGGATGACGTCAAGTCATC

ATGGCCCTTACGGCCTGGGCTACACACGTGC

>Pseudomonas_monteilii_ZAAgR1_(PX904367)

ACATGCAAGTCGAGCGGATGACGGGAGCTTGCTCCTTGATTCAGCGGCGGACGGGTGAGTAATGCCTAGGAATCTGCCTGGTAGTGGG

GGACAACGTTTCGAAAGGAACGCTAATACCGCATACGTCCTACGGGAGAAAGCAGGGGACCTTCGGGCCTTGCGCTATCAGATGAGCC

TAGGTCGGATTAGCTAGTTGGTGGGGTAATGGCTCACCAAGGCGACGATCCGTAACTGGTCTGAGAGGATGATCAGTCACACTGGAAC

TGAGACACGGTCCAGACTCCTACGGGAGGCAGCAGTGGGGAATATTGGACAATGGGCGAAAGCCTGATCCAGCCATGCCGCGTGTGTG

AAGAAGGTCTTCGGATTGTAAAGCACTTTAAGTTGGGAGGAAGGGCAGTAAGTTAATACCTTGCTGTTTTGACGTTACCGACAGAATA
AGCACCGGCTAACTCTGTGCCAGCAGCCGCGGTAATACAGAGGGTGCAAGCGTTAATCGGAATTACTGGGCGTAAAGCGCGCGTAGGT

GGTTCGTTAAGTTGGATGTGAAAGCCCCGGGCTCAACCTGGGAACTGCATCCAAAACTGGCGAGCTAGAGTACGGTAGAGGGTGGTGG

AATTTCCTGTGTAGCGGTGAAATGCGTAGATATAGGAAGGAACACCAGTGGCGAAGGCGACCACCTGGACTGATACTGACACTGAGGT

GCGAAAGCGTGGGGAGCAAACAGGATTAGATACCCTGGTAGTCCACGCCGTAAACGATGTCAACTAGCCGTTGGAATCCTTGAGATTT

TAGTGGCGCAGCTAACGCATTAAGTTGACCGCCTGGGGAGTACGGCCGCAAGGTTAAAACTCAAATGAATTGACGGGGGCCCGCACAA

GCGGTGGAGCATGTGGTTTAATTCGAAGCAACGCGAAGAACCTTACCAGGCCTTGACATGCAGAGAACTTTCCAGAGATGGATTGGTG

CCTTCGGGAACTCTGACACAGGTGCTGCATGGCTGTCGTCAGCTCGTGTCGTGAGATGTTGGGTTAAGTCCCGTAACGAGCGCAACCCT

TGTCCTTAGTTACCAGCACGTAATGGTGGGCACTCTAAGGAGACTGCCGGTGACAAACCGGAGGAAGGTGGGGATGACGTCAAGTCAT

CATGGCCCTTACGGCCTGGGCTACACACGTGCTACAATGGTCGGTACA

>Acinetobacter_junii_ZB7_(PX904041)

ATGCAAGTCGAGCGGAGATGAGGTGCTTGCACCTTATCTTAGCGGCGGACGGGTGAGTAATGCTTAGGAATCTGCCTATTAGTGGGGG

ACAACATTCCGAAAGGAATGCTAATACCGCATACGTCCTACGGGAGAAAGCAGGGGATCTTCGGACCTTGCGCTAATAGATGAGCCTA

AGTCGGATTAGCTAGTTGGTGGGGTAAAGGCCTACCAAGGCGACGATCTGTAGCGGGTCTGAGAGGATGATCCGCCACACTGGGACTG

AGACACGGCCCAGACTCCTACGGGAGGCAGCAGTGGGGAATATTGGACAATGGGGGGAACCCTGATCCAGCCATGCCGCGTGTGTGA

AGAAGGCCTTATGGTTGTAAAGCACTTTAAGCGAGGAGGAGGCTACTGAGACTAATACTCTTGGATAGTGGACGTTACTCGCAGAATA

AGCACCGGCTAACTCTGTGCCAGCAGCCGCGGTAATACAGAGGGTGCGAGCGTTAATCGGATTTACTGGGCGTAAAGCGTGCGTAGGC

GGCTTTTTAAGTCGGATGTGAAATCCCCGAGCTTAACTTGGGAATTGCATTCGATACTGGGAAGCTAGAGTATGGGAGAGGATGGTAG

AATTCCAGGTGTAGCGGTGAAATGCGTAGAGATCTGGAGGAATACCGATGGCGAAGGCAGCCATCTGGCCTAATACTGACGCTGAGGT

ACGAAAGCATGGGGAGCAAACAGGATTAGATACCCTGGTAGTCCATGCCGTAAACGATGTCTACTAGCCGTTGGGGCCTTTGAGGCTT

TAGTGGCGCAGCTAACGCGATAAGTAGACCGCCTGGGGAGTACGGTCGCAAGACTAAAACTCAAATGAATTGACGGGGGCCCGCACA

AGCGGTGGAGCATGTGGTTTAATTCGATGCAACGCGAAGAACCTTACCTGGCCTTGACATACTAGAAACTTTCCAGAGATGGATTGGT

GCCTTCGGGAATCTAGATACAGGTGCTGCATGGCTGTCGTCAGCTCGTGTCGTGAGATGTTGGGTTAAGTCCCGCAACGAGCGCAACCC

TTTTCCTTACTTGCCAGCATTTCGGATGGGAACTTTAAGGATACTGCCAGTGACAAACTGGAGGAAGGCGGGGACGACGTCAAGTCAT

CATGGCCCTTACGGCCAGGGCTACACACGTGCTACAATGGTCGGTACAAAGGGTTGCTACACAGCGATGTGATGCTAATCTC

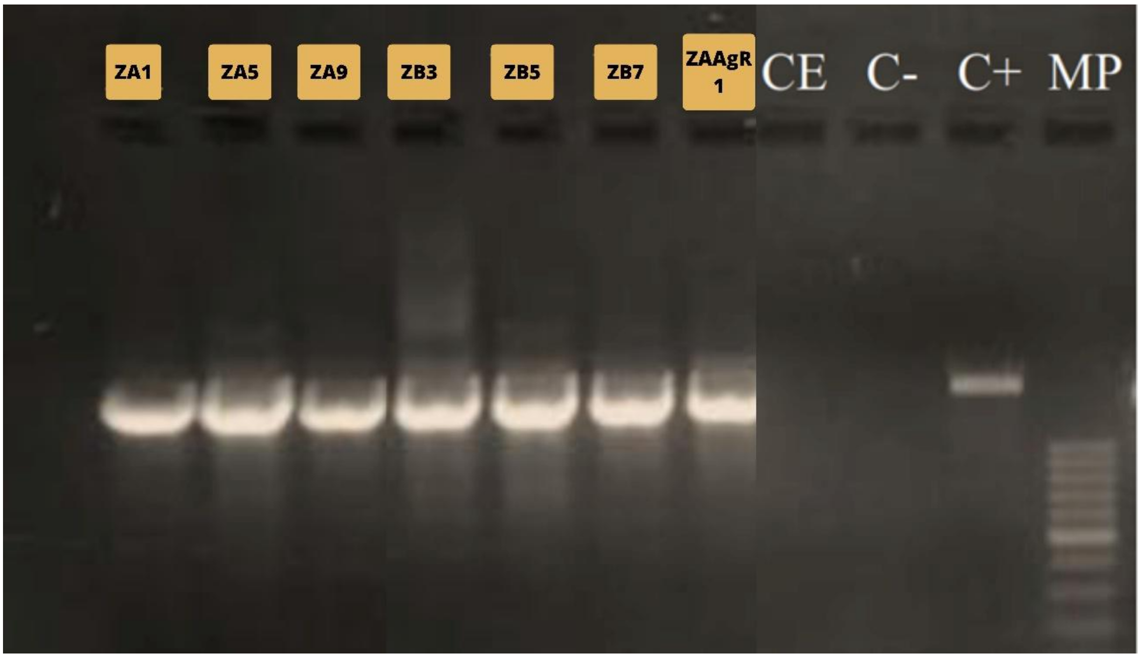

Fig. 1. Gen 16SrRNA by horizontal electrophoresis in a 1.5% agarose gel. C+: positive control (*B. cereis)*, C-: negative control (AUP) y CE (AUP treated).
